# Effect of cetrimonium carrier micelles on bacterial membranes and extracellular DNA, an in silico study

**DOI:** 10.1038/s41598-023-32475-x

**Published:** 2023-05-17

**Authors:** Jhonatan Soto Puelles, Mahdi Ghorbani, Benjamin Tuck, Laura L. Machuca, M. Leigh Ackland, Fangfang Chen, Anthony E. Somers, Maria Forsyth

**Affiliations:** 1 Institute for Frontier Materials, Deakin University, Geelong, VIC 3217 Australia; 2 Curtin Corrosion Centre, WA School of Mines: Minerals, Energy and Chemical Engineering, Curtin University, Kent Street, Bentley, WA 6102 Australia; 3 ARC Centre of Excellence for Electromaterials Science (ACES), Deakin University, Burwood, 3125 Australia; 4 School of Life and Environmental Sciences, Deakin University, Burwood, Victoria 3125 Australia

**Keywords:** Structural biology, Cell death, DNA, Computational chemistry

## Abstract

Microorganisms do not live as dispersed single cells but rather they form aggregates with extracellular polymeric substances at interfaces. Biofilms are considered efficient life forms because they shield bacteria from biocides and collect dilute nutrients. This is a big concern in industry since the microorganisms can colonize a wide range of surfaces, accelerating material deterioration, colonizing medical devices, contaminating ultrapure drinking water, increasing energy costs and creating focus of infection. Conventional biocides that target a specific component of the bacteria are not effective in the presence of biofilms. Efficient biofilm inhibitors are based on a multitarget approach interacting with the bacteria and the biofilm matrix. Their rationale design requires a thorough understanding of inhibitory mechanisms that are still largely lacking today. Herein we uncover via molecular modelling the inhibition mechanism of cetrimonium 4-OH cinnamate (CTA-4OHcinn). Simulations show that CTA-4OH micelles can disrupt symmetric and asymmetric bilayers, representative of inner and outer bacterial membranes, following three stages: adsorption, assimilation, and defect formation. The main driving force for micellar attack is electrostatic interactions. In addition to disrupting the bilayers, the micelles work as carriers facilitating the trapping of 4OH cinnamate anions within the bilayer upper leaflet and overcoming electrostatic repulsion. The micelles also interact with extracellular DNA (e-DNA), which is one of the main components of biofilms. It is observed that CTA-4OHcinn forms spherical micelles on the DNA backbone; which hinders their ability to pack. This is demonstrated by modelling the DNA along the hbb histone-like protein, showing that in the presence of CTA-4OHcinn, DNA does not pack properly around hbb. The abilities of CTA-4OHcinn to cause cell death through membrane disruption and to disperse a mature, multi-species biofilm are also confirmed experimentally.

## Introduction

Biofilms are dynamic, complex networks of microbial aggregates formed by microorganisms and extracellular polymeric substances (EPS); including extracellular DNA (eDNA), carbohydrates, proteins and other organic and inorganic compounds^1–5^. They provide protection to the bacteria and help them proliferate under certain conditions^6^. Around 40–80% of bacteria can form biofilms on natural and synthetic surfaces, and in several scenarios, they represent a health risk. For instance, they can grow on medical devices, prosthetics and patient tissues^7^; they also can be found on the inner surfaces of metal pipes of hospital water systems^8^. Biofilm-related organisms are responsible for at least 65% of all microbial infections^9^ and they are commonly associated with cystic fibrosis, chronic wounds, otitis media, endocarditis and osteomyelitis^10^. Biofilms also represent a critical problem in the food industry, since some bacteria easily proliferate in food processing environments due to the availability of carbon sources. They account for 60% of foodborne outbreaks threatening human health and resulting in severe economic losses^11,12^. Industrial metals such as copper, aluminum and iron/steel are also susceptible to biofilm formation, which in some cases can accelerate its deterioration. Such phenomena is known as microbiologically influenced corrosion (MIC) and it accounts for at least 20% of the total corrosion cost, estimated to be around $2.5 trillion annually^13,14^.

Unlike planktonic bacteria, which are in a free-living state, biofilms are persistent against ordinary disinfection methods^15,16^. EPS holds bacteria together and provide a microenvironment within the biofilm, forming an effective barrier against altered pH, nutrient scarcity, shear forces, antibiotics and host immune cells^17,18^. Classical antibiotic therapy cannot completely eradicate bacteria located within the biofilm and alternative methods such as nanoparticles, matrix-degrading enzymes and anti-quorum sensing signaling molecules have been studied^19^. These compounds target specific components of the biofilm; for example, DNase, a DNA degrading enzyme, attacks eDNA causing biofilm dispersion^20^. Even with the use of advanced analytical techniques, the mechanism of interaction of a potential biofilm-inhibitor is not clear, which hinders its rational design and optimization.

Molecular modelling (MM) could provide an efficient solution to understand underlying mechanisms that lead to an effective inhibition and elimination of biofilms. MM has a critical role in understanding the interaction of antimicrobials with bacterial membranes and has previously been used to identify a number of different mechanisms through which compounds act on bacterial membranes. Orekhov et al. used coarse grained simulations to show how the phthalocyanine ZnPcChol^8+^ has an affinity for the inner and outer membrane of gram-negative bacteria and reaches an energetically favorable configuration when inserted within the bilayers, thus increasing the bacteria wall permeability^21^. Another study showed that the antimicrobial peptide (AMP) Magainin-2 has an affinity to bind liquid-disordered regions on a bilayer and dimerize in agreement with experimental data. A higher concentration of the AMP resulted in the spontaneous formation of toroidal pores confirming the membranolytic activity of Magainin-2^22^. Interactions of the AMP Polymyxin B1 with the inner and outer membrane of *E. coli* were modelled and showed that the lipopolypeptide forms aggregates on the outer membrane surface, but no insertion occurred. Conversely, Polymyxin B1 easily inserted in the inner membrane causing its destabilization since the hydration within the bilayer increased^23^. Simulations on another AMP, BP-100, showed that the antimicrobial initially bound to the bilayer via its hydrophilic residues, following which the BP-100 flipped with its hydrophobic residues facing the inner core of the membrane^24^.

In the present work, we used coarse-grained and all-atom molecular modelling to elucidate the interaction mechanism between the surfactant cetrimonium 4-OH cinnamate (CTA-4OHcinn), which is a non-toxic compound with anti-microbial and corrosion inhibition properties^25–28^, and different components of a biofilm such as bacterial membranes, eDNA and chromatin. Firstly, the effect of the inhibitor on inner bacterial membranes was modelled by constructing symmetric bilayers composed of palmitoyloleoyl phosphatidylethanolamine (POPE) and palmitoyloleoyl phosphatidylglycerol (POPG). POPE and POPG are the main components of inner bacterial membranes^29^. Following this the effect of CTA-4OHcinn on asymmetric bilayers characteristic of gram-negative bacterial outer membranes was studied. For this section, Re-LPS was chosen from *Escherichia coli* to model the bilayers upper leaflet since the forcefield parameters for Re-LPS are available. regarding the interaction between CTA-4OHcinn and bacterial chromatin, cytosine–guanine and adenine–thymine DNA chains were modelled as a representative of the e-DNA lattice of biofilms. Additionally, the effect of the inhibitor on DNA packing within bacteria chromatin was modelled by considering the interaction between the bacterial protein hbb and DNA. Hbb was selected since its molecular mechanism is already known^30^. Previous research regarding the interaction between micellar systems and bacterial membranes or chromatin are limited; and although there are many proposed mechanisms regarding how micelles attack bacteria^31,32^, to our knowledge this is the first modelling study that demonstrates at a molecular detail the impact of cationic micelles on different parts of the biofilm.

## Methods

The present work is divided in two sections. In the first part, the interaction between CTA-4OHcinn and bacterial membranes is studied. CTA-4OHcinn and membrane interactions were simulated using the Polarizable Water Martini Coarse-Grained model (PW-Martini)^33^. The symmetric bilayers were composed of palmitoyloleoyl phosphatidylethanolamine (POPE) and palmitoyloleoyl phosphatidylglycerol (POPG) lipids since these are the main components of inner bacterial membranes^29^. Their molecular structure along with their coarse-grained representation is shown in Fig. S-1. Preassembled bilayers were constructed using the MartiniMaker tool from the CHARMM-GUI interface^34–36^. Validation of POPE and POPG bilayers was done by comparing the predicted bilayer thickness and area per lipid (APL) with their respective experimental counterparts, as detailed in Supplementary Information (Fig. S-3). For modelling the micelle-bilayer interaction, the setup consisted of two bilayers enclosing a micelle. Similar compartmentalization was used in modelling transmembrane potential gradients^37^ and allows to separate an inner and outer phase for further analysis. The initial setup is shown in Fig. S-4. The modelling of the micelles is detailed in S.I.

Since the original PW Martini model does not use long range interactions^33^, we considered that leaving a space of 8 nm between the bilayers in the outer space region was enough to ensure no-interaction between both bilayers. This was further supported by showing no considerable variation in the thickness of a 58% POPE + 42% POPG bilayer in a single and double bilayer setup (Fig. S-3i and the dotted curve in Fig. S-12b, respectively).

To simulate the outer membrane of a gram-negative bacteria, whose upper leaflet is formed by lipopolysaccharides (LPS), the Re-LPS from *E. coli* was considered since its parametrization in PW-Martini is available in CHARMM-GUI^35^. Re-LPS is an incomplete molecule that lacks the O-antigen, also known as rough LPS. Bacteria that contain rough LPS in their outer membrane are more susceptible to antibiotics^38^ and we opted for this model to optimize our computational resources, as was conducted previously^23^. The study of an outer membrane with smooth LPS (LPS with the O antigen) can be considered in future work. Details of the CG model for Re-LPS are discussed in S.I., Fig. S-2.

Although divalent cations such as Ca^2+^ should be considered in the outer membrane model^39,40^ we opted for using Na^+^, since PW-Martini standard setup uses short range electrostatic interactions via the Reaction Field algorithm, which is not accurate at predicting small ion interactions. The Martini 2.0 library warns that calcium cations are untested, and are likely to give an unrealistic description. Other studies have changed the simulation setup to predict the behavior of divalent cations, for example using a Particle-mesh Ewald summation instead of a Reaction Field, but changing the setup also affects the interaction of the other molecules and requires reparameterization^41,42^.

Coarse-grained (CG) parameters for cetrimonium ions were taken from previous studies, whose model was validated with experimental SAXS data^43,44^. The cetrimonium CG parameters were tested and compared with the OPLS-AA and CHARMM all-atom forcefields, as discussed in S.I. Fig. S-5. Regarding 4-OH cinnamate, a bottom-up approach was adopted for constructing its parameters beginning from all-atom parameters previously published^26^. A detailed discussion and validation of the 4-OH cinnamate CG model can be found in S.I., Figs. S-6 and S-7. Simulation details are provided in the Supplementary Information.

For the second part, the interaction between CTA-4OHcinn and bacterial chromatin was studied. DNA simulations were conducted using the CHARMM all-atom forcefield. Firstly, we created two types of DNA molecules, each containing 16 base pairs using the NAFlex web server^45^. The first one was composed of only adenine–thymine pairs (AT-DNA) and the second contained only cytosine–guanine pairs (CG-DNA). The initial structures were solvated and neutralized with the CHARMM-GUI interface^36^. Both DNA molecules were simulated for at least 100 ns. Simulation details and validation are provided in S.I., Figs. S-8, and S-9. The histone-like protein Hbb from *Borrelia burgdorferi* was chosen to model the effect of CTA-4OHcinn on the DNA–protein packaging. The molecular structure of Hbb, determined by X-ray diffraction experiments^46^, can be found in The Protein Data Bank web server^47^. Since DNA and Hbb will be in the same system, the CHARMM forcefield was also used for the histone-like protein, with parameters taken from the CHARMM-GUI interface^36^. A detailed discussion can be found in S.I., Fig. S-10. All simulations and post-analyses were performed using the molecular modelling engine Gromacs 2020.3^48^ and trajectory visualization was obtained with VMD^49^. Table S-1 shows a summary of the simulations.

Regarding the experimental section, the synthesis of CTA-4OHcinn is detailed in our previous work^28^. Confocal laser scanning microscopy (CLSM) was performed on *Klebsiella pneumoniae *(a gram-negative encapsulated bacillus) before and after 10 mM cetrimonium 4-OH cinnamate treatment. The isolate was chosen for its thick outer capsule and growth rate in seawater. This sample was concentrated and cleaned in the log phase by centrifugation at 8k rpm for 5 min before resuspending the pellet in sterile phosphate-buffered saline (PBS; Sigma, pH 7.4). Healthy bacterial cells were acclimatized for 1 h at 30 °C before combining with 10 mM CTA-4OHcinn (experimental) and Cellbrite™ Fix 555 in a 10 × final concentration. A Nikon A1 + confocal microscope equipped with a 100 × objective was used for all imaging.

CLSM was also employed to visualize the attachment of eDNA to carbon steel (CS) after treatment of a healthy biofilm with 10 mM CTA-4OHcinn. Mature biofilms were stained with propidium iodide (Invitrogen™) for 10 min before and after 4 h of treatment with 10 mM CTA-4OHcinn dissolved in PBS (Sigma, pH 7.4) as described previously^50^. Biofilms were developed using the following bacterial strains, inoculated into Centre for Disease Control (CDC) bioreactors and evaluated after 2 weeks of growth: *Pseudomonas balearica* EC28, *Shewanella chilikensis* DC57 and a laboratory strain of *K. pneumoniae*, as previously described^50^. CLSM was conducted using a 20 × dry objective lens, with propidium iodide signal captured in sequential micrographs using a 561 nm laser and 570–620 nm emission filter.

## Results and discussion

### Effect of CTA-4OHcinn on symmetric and asymmetric bilayers

**Figure 1:**
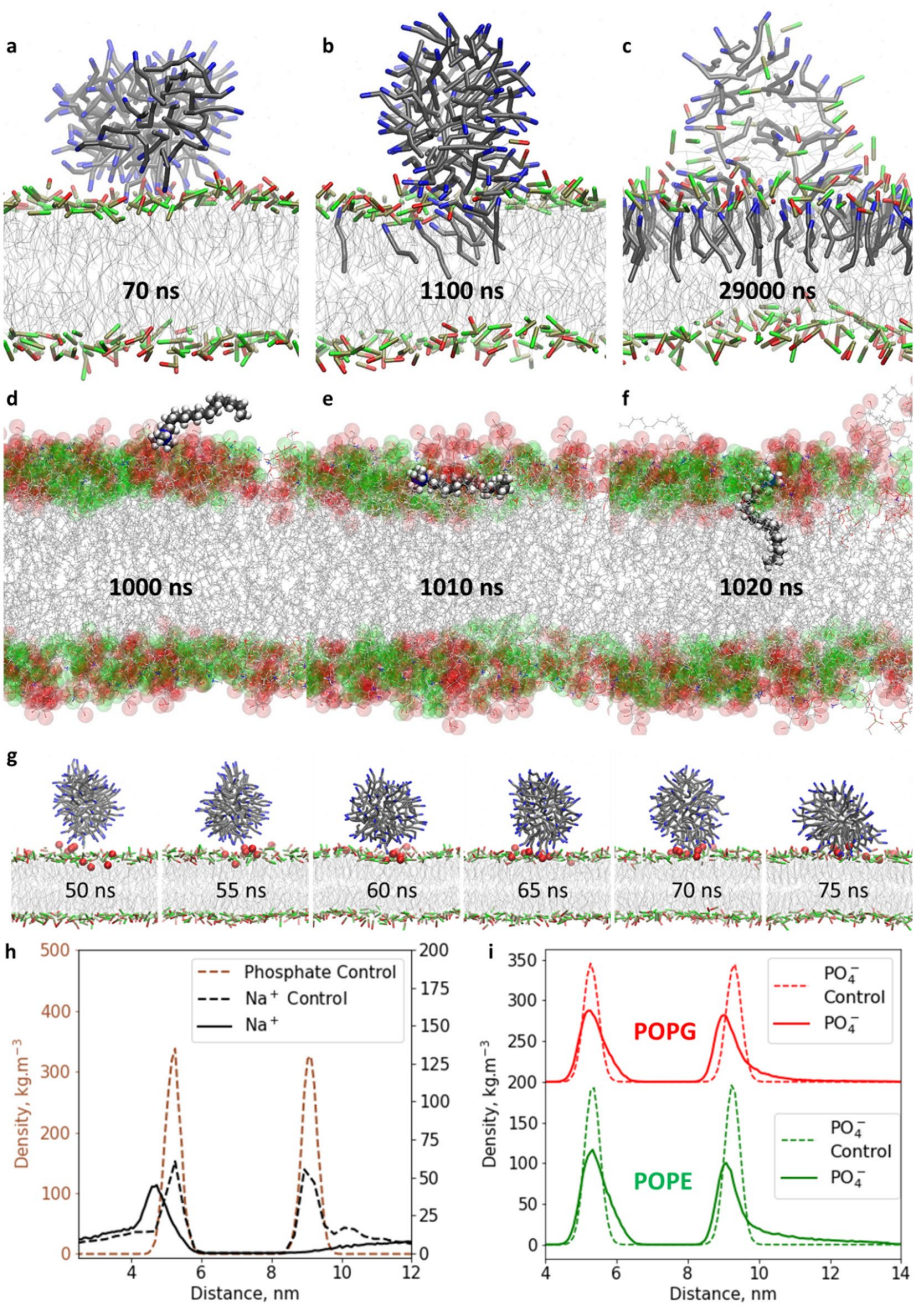
Simulation snapshots of a cetrimonium micelle with chloride counterions and a bilayer composed of 52% POPE + 48% POPG. Note water, Na and Cl counterions are not depicted. POPE and POPG polar heads are green and red, respectively; and the cetrimonium amine groups are blue (**a**–**c**). Cetrimonium insertion mechanism in all atom representation obtained with a backmapping algorithm^55^ (**d**–**f**). Segregation of POPG amine heads (red spheres) as a result of micelle attachment on the mixed bilayer (**g**). Density profiles of phosphate groups and sodium counterions along the direction normal to the bilayer; the peaks at 5 and 9 nm represent the lower and upper leaflet respectively and the dashed lines correspond to the control simulation without micelle (**h**). Density profile of POPE and POPG polar heads before (control) and after cetrimonium interaction where POPG profile has been shifted upward 200 units for visualization purposes (**i**).

At first, we studied the interaction between a micelle consisting of cetrimonium cations and chloride counterions and a mixed bilayer composed of 52 mol% POPE and 48 mol% POPG (System s12 from Table S-1), which is the representative composition of the inner membrane from *Pseudomonas aeruginosa*^51^. MD simulations showed that micelle fusion occurs following three stages, as shown in Fig. 1. The micelle was firstly adsorbed on the bilayer surface due to electrostatic interactions between the positive micelle outer shell, formed by cetrimonium amine groups, and the negative POPG polar heads, as illustrated in a snapshot taken at 70 ns in the simulations in Fig. 1a. We confirmed that electrostatic interactions were the main driving force for micelle adsorption through additional simulations between a cetrimonium micelle and a bilayer composed of neutral POPE, which did not present the same significant interaction, as is shown in S.I., Fig. S-11. In the second stage, the adsorbed micelle began to inject cetrimonium molecules into the bilayer upper leaflet, as seen at 1100 ns in the simulation in Fig. 1b. Visual inspection of this injection process in Fig. 1d–f showed that the integration of cetrimonium molecules followed a flip mechanism, where the amine group was electrostatically bonded with the negative POPG phosphate group, and the cetrimonium alkyl chain was initially protruding from the bilayer (Fig. 1d). As a result of Van der Waals interactions, the cetrimonium alkyl chain is then positioned parallel to the bilayer surface and eventually, is flipped downward, integrating with the bilayer (Fig. 1e,f). A similar flip mechanism was reported regarding the insertion of the antimicrobial polypeptide BP-100 in a 1,2-dipalmitoyl-sn-glycerol-3-phosphoglycerol (DPPG) bilayer, where the hydrophobic residues of BP-100 were buried within the bilayer due to Van der Waals interactions^24^. In the third stage (29,000 ns in Fig. 1c), once the micelle has merged within the bilayer, a lump defect is created with a mixture of cetrimonium, POPE and POPG molecules. As the micelle approached the bilayer surface, POPG segregation occurred, creating a temporal local zone with enough electrostatic attraction to drive the adsorption event (Fig. 1g). This is confirmed by a top view snapshot of the micelle bound to the bilayer surface (Fig. S-15a). As a result of the integration of the cetrimonium molecules within the bilayer’s upper leaflet, the net negative charge was reduced, weakening the electrostatic attraction between the negative bilayer surface and its sodium counterions, causing the displacement of the latter (Fig. 1h at 9 nm). This was supported by simulation snapshots before and after micelle fusion showing a considerable decrease in sodium counterions after bilayer disruption (Fig. S-18a,b). Additionally, POPE diffuses more easily within the lump defect compared to POPG since its profile extends further out from the main membrane surface peak than POPG (14 nm and 12 nm, respectively), as can be seen in their density profiles taken from the last microsecond (Fig. 1i). The lower quantity of POPG compared to POPE within the lump might also be the result of the lower mobility of POPG in the bilayer. It was also noticed that chloride counterions were electrostatically repelled from the bilayer surface and after micelle fusion, as well as being absent within the bilayer’s upper leaflet (Fig. S-12).

The simulation is consistent with the micelle fusion and membrane disruption mechanism observed between alkylphospholipids and carcinogenic cells^31^. Surfactant inclusion within phospholipid membranes was also shown experimentally for the biosurfactant trehalose that could incorporate within dimyristoyl phosphatidylserine (DMPS) membranes, decreasing lipid mobility^52^. The mechanism further proposes that the bilayer is completely disrupted, forming micelles composed of the cationic surfactants and bilayer phospholipids^53^. This final state could not be reached due to the limitation in simulation time in this work. It is likely that as a result of the defect formed by cetrimonium, cellular stress will build upon the membrane promoting cell lysis^31^. It was reported that cetrimonium bromide at sub-micellar concentrations was able to reduce the viability of *Candida albicans*; however, rather than lysis, a change in the cell surface charge was proposed as the dominant mechanism^54^.

Previous experimental and modelling studies have shown that CTA-4OHcinn forms distinct micelles where the 4-OH cinnamate anions are integrated within the cetrimonium micelles in an oriented way with their carboxylates located in the outer shell and their hydroxyls deeper inside the micelle^25,26^. Therefore, to study the effect of 4OH cinnamate on membrane disruption, a CTA-4OHcinn micelle was simulated above a mixed bilayer with a similar composition to the previous bilayer; i.e. 52% POPE + 48% POPG (System s15 from Table S-1). It was noticed that the adsorption of the CTA-4OHcinn micelle on the bilayer was much slower, occurring at 15 µs, compared to the cetrimonium chloride micelle, which only needs tens of nanoseconds. This could be the result of the embedded 4-OH cinnamate anions within the micelle that reduce its effective positive charge and therefore its electrostatic attraction with the bilayer surface was weakened. Just before micelle adsorption, a local zone with a higher composition of POPG lipids was also observed, corresponding to POPG segregation (Figs. S-13a–d and S-15b).The adsorption was unstable and eventually, the micelle was detached leaving some inserted cetrimonium molecules in the bilayer upper leaflet. Cetrimonium insertion followed the flip mechanism previously described (Fig. S-13e). Micelle fusion was not observed within the simulation time (30 µs).

**Figure 2:**
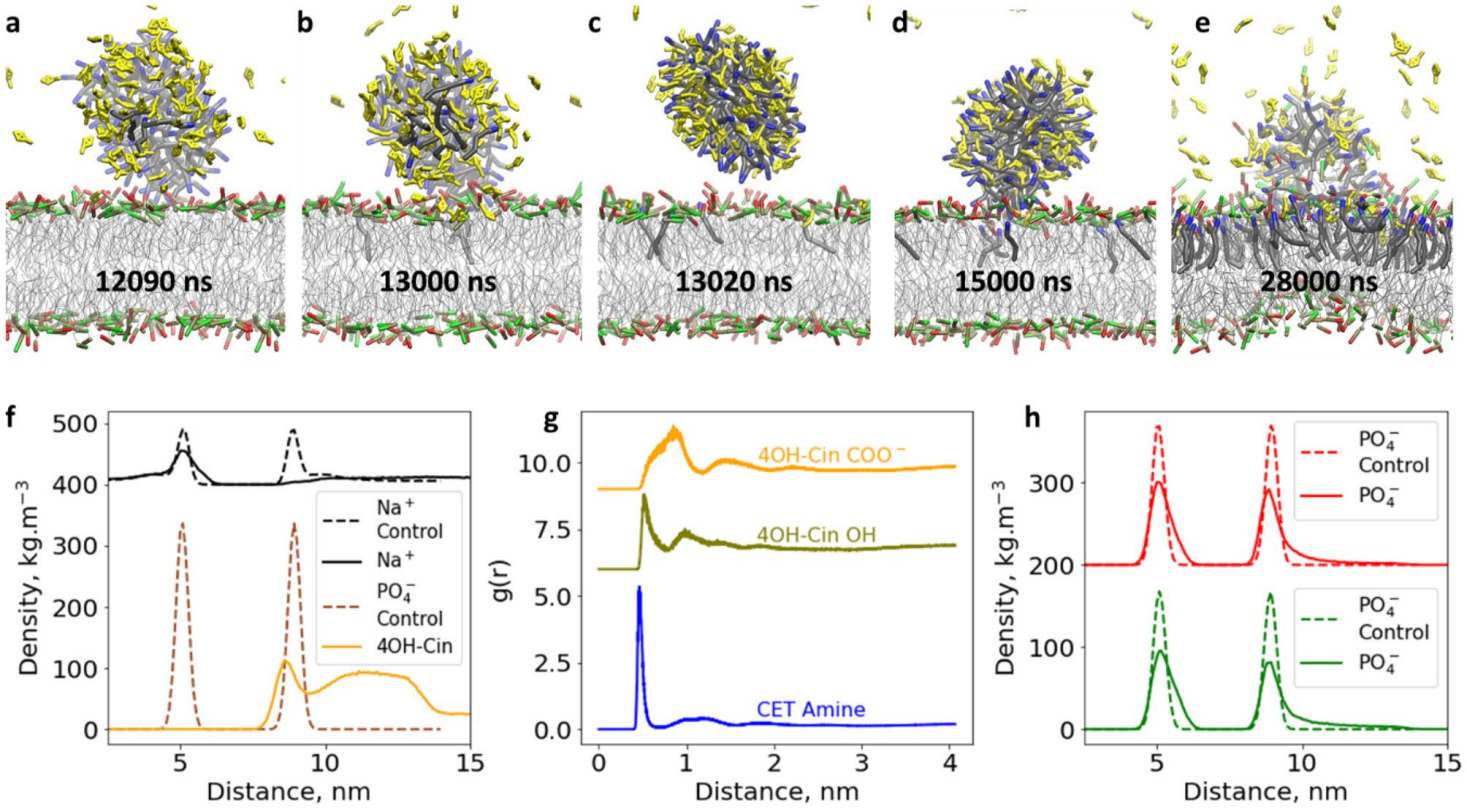
Simulation snapshots of a CTA-4OHcinn micelle and a mixed bilayer composed of 50% POPE + 50% POPG. Cetrimonium amine groups and 4-OH cinnamate anions are blue and yellow, respectively (**a**–**e**). Density profile in the normal direction from the bilayer surface for sodium counterions, lipid phosphates and 4-OH cinnamate; the peaks at 5 and 9 nm represent the lower and upper leaflet respectively and the dashed lines correspond to the control simulation without micelle (**f**). RDF for the carboxylate and hydroxyl groups from 4-OH cinnamate and cetrimonium amine group taking as a reference the phosphate polar head from the lipids. The hydroxyl and carboxylate curves were shifted upward 6 and 9 units, respectively (**g**). Density profile of POPG and POPE phosphate heads, before and after 4-OH cinnamate loaded cetrimonium micelle interaction where POPG profile has been shifted upward 200 units for visualization purposes (**h**).

Since it was demonstrated that electrostatic interactions are the main driving force for micelle adsorption, the negative net charge of the bilayer surface that mainly comes from POPG was increased by slightly raising the POPG proportion from 48 to 50% (System s19 from Table S-1). Two adsorption events were captured within the simulated 30 µs. The first one occurred at around 12 µs and similar to the previous case, the micelle was detached from the bilayer surface after 1 µs (Fig. 2a–c), leaving some injected cetrimonium molecules via the same flip mechanism in the bilayer (Fig. S-14). A second adsorption event occurred at around 15 µs and this time the micelle was attached to the bilayer surface more stably. The adsorbed micelle provided enough time for the insertion of more cetrimonium molecules and eventually merged with the bilayer (Fig. 2d,e).

Although it is shown that the CTA-4OHcinn micelle takes longer to disrupt the bilayer, contrary to the cetrimonium chloride micelle; this time, the counterions (i.e. 4OH cinnamate) were not repelled by the bilayer surface and they were trapped within the bilayer upper leaflet, overcoming electrostatic repulsion (Fig. 2f). The accumulation of 4OH cinnamate in the bacteria wall might contribute to the biocidal effect of CTA-4Ohcinn; since 4OH cinnamate was reported as a natural antibacterial compound^56^. The trapped 4-OH cinnamate anions, carried by the micelle, within the upper leaflet followed a certain orientation, where their hydroxyl group was found deeper compared to the carboxylate group (Fig. 2g). Such orientation might be the result of a strong electrostatic interaction between the POPE amino group and the carboxylate from the aromatic anion. The right-skewed distribution of the density profile for the POPG and POPE polar heads (Fig. 2h) suggests the deformation of bilayer lipids due to formation of the lump defect, which could eventually detach from the bilayer, creating a micelle composed of lipids^53,57^. The synergy of the CTA-4OHcinn micelle disrupting the bilayer was demonstrated by performing additional simulations between 4-OH cinnamate with sodium counterions and the same bilayer (System s20 from Table S-1). As shown in S.I. 4-OH cinnamate was not adsorbed considerably likely due to an electrostatic repulsion with the bilayer surface (Fig. S-16). This simulation (Fig. S-16) suggests that below the inhibitor critical micelle concentration, the 4OH-cinnamate anions will not be adsorbed on the bilayer surface since there are no carrier cetrimonium micelles. Regarding cetrimonium monomers, they might be adsorbed on the bilayer due to electrostatic interactions and further incorporated. Eventually, there will be enough inserted cetrimonium molecules within the upper leaflet to create a defect, however this is likely to be slower and less effective than when the large micelles arrive at a membrane.

Following the simulations of symmetric bilayers that might represent the plasma membrane of a gram-positive bacteria or the inner membrane of a gram-negative bacteria, the interaction between a CTA-4OHcinn micelle and asymmetric bilayers were considered, representative of a gram negative bacterial outer membrane. A mature bacteria might have LPS covering 75% of its outer surface^58^, the rest being proteins and other lipids. Based on the proposed biosynthetic pathway for LPS, the assembly of the lipid section and the O-antigen, composed of oligosaccharides, occurs in the space between the inner and outer membrane, known as the periplasmic space. Once the LPS is assembled, it is exported from the lower leaflet to the bacteria surface via a flip-flop mechanism, where some phospholipids are substituted by the respective LPS^59,60^. Based on this mechanism, we assume that at an early stage or due to some disruption event, the bacteria will have a depletion of LPS in its outer surface. Since the lower leaflet is mostly composed of POPE and POPG with varying proportions^61,62^, we also assumed that the LPS-depleted regions should also contain the same type of lipids.

Initially an asymmetric bilayer with an upper leaflet composed of 75% Re-LPS and 25% POPE was modelled (System s23 from Table S-1). Simulations showed that the CTA-4OHcinn micelle was adsorbed within the first 200 ns, due to a strong electrostatic interaction between the cetrimonium amine groups and the negative bilayer surface, since each molecule of Re-LPS has a charge of − 6 (Fig. S-17a). As observed in the density profile, the bilayer thickness and the adsorbed sodium counterions were not affected upon micelle adsorption (Fig. S-17d). Additionally, a few 4-OH cinnamate anions, carried within the composed micelle, penetrated the Re-LPS hydrophilic head reaching the phosphate groups and overcoming electrostatic repulsion between the anions and the upper leaflet. The adsorbed sodium counterions on the bilayer surface might bridge the negative Re-LPS heads, forming an effective barrier against micelle intrusion^23,63^. As the Re-LPS concentration was decreased to 50% and 25% in the upper leaflet (Systems s25 and s24, respectively from Table S-1), there was a decrease in the adsorbed sodium counterions (Fig. S-17e,f) since the bilayer surface was less negative and therefore the attraction force decreased. It is likely that electrostatic attraction between the oligosaccharide portion of LPS and the cetrimonium amine groups is the main driving force for the micelle adsorption on the asymmetric bilayer surface since the previous simulation between the micelle and a pure POPE bilayer showed no micelle binding (Fig. S-11). The increase of trapped 4OH-cinnamate anions within the upper leaflet at higher POPE concentration (Fig. S-16d–f) indicates that once the micelle is anchored to the bilayer surface, it begins to release its aromatic counterions that overcome the electrostatic repulsion of the LPS oligosaccharides and reach the phosphate groups. The presence of trapped 4OH-cinnamate in the upper leaflet, which is highly negative, demonstrates the carrier role of the inhibitor micelle in bringing active negative agents within the bilayer. The absence of trapped 4OH-cinnamate in the pure POPE bilayer (Fig. S-11) shows that, without the oligosaccharides from LPS providing an anchor point for the cetrimonium cation that forms the micelle, the 4OH-cinnamate anion is not delivered in close enough proximity to reach the phosphate groups.

It is also noticed that at 25% Re-LPS concentration, some cetrimonium molecules were inserted within the bilayer’s upper leaflet following the already observed flip mechanism; however, no micelle merging occurred throughout the simulation (Fig. S-17g).

**Figure 3:**
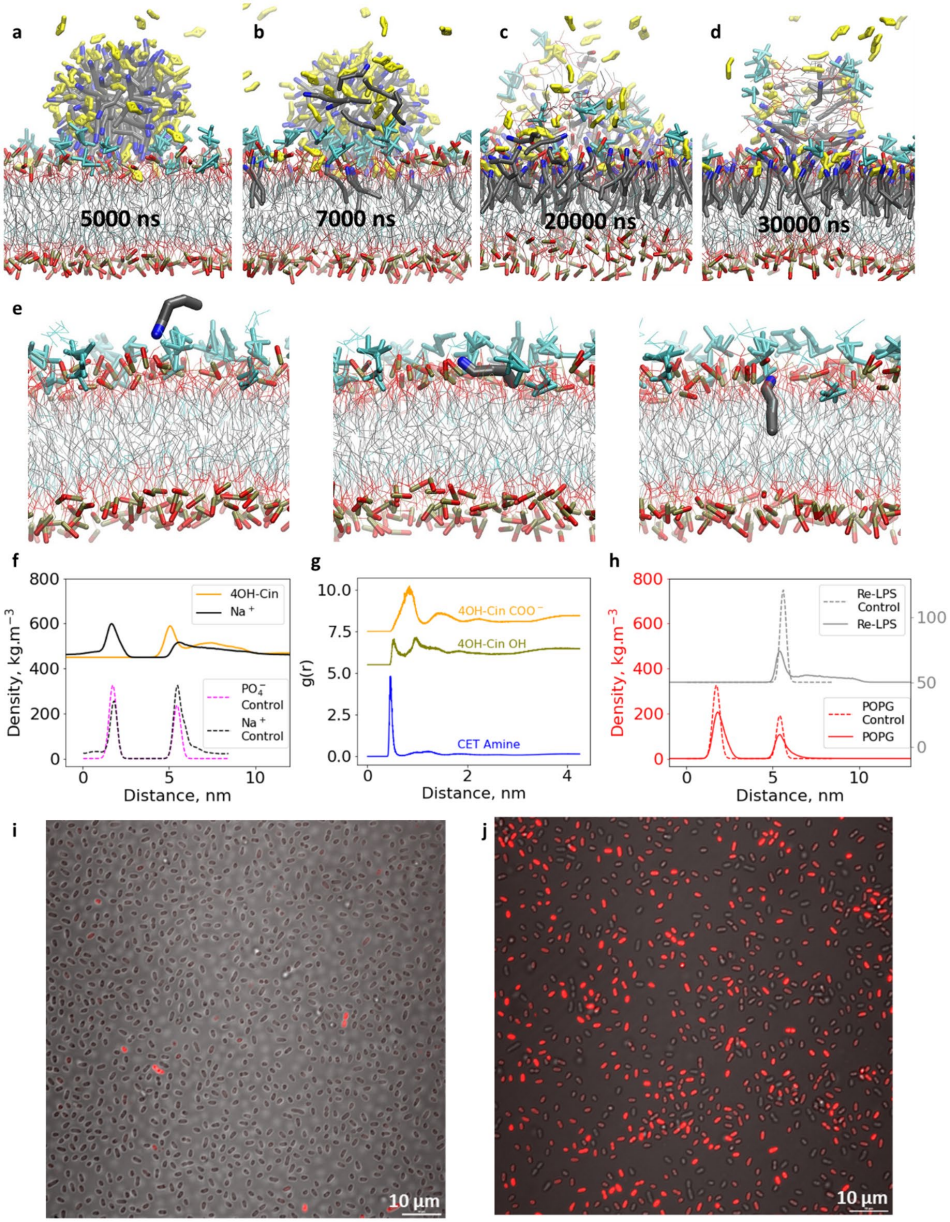
Simulation snapshots showing the interaction between a CTA-4OHcinn micelle and an asymmetric bilayer composed of 25% Re-LPS + 75% POPG in the upper leaflet and 100% POPG in the lower leaflet (**a**–**d**). Flip mechanism for cetrimonium insertion (**e**). Density profile of sodium counterions and 4-OH cinnamate anions (shifted upward by 450 units) along with their respective control simulations (without micelle interaction) (**f**). RDF for the carboxylate and hydroxyl groups from 4-OH cinnamate and cetrimonium amine group taking as a reference the phosphate polar head from the lipids. The hydroxyl and carboxylate curves were shifted upward 5.5 and 7.5 units, respectively (**g**). Density profile of POPG and LPS phosphate heads, before and after micelle interaction, LPS profile has been shifted upward 350 units for visualization purposes (**h**). Confocal micrographs of *K. pneumoniae* before and after treatment with 10 mM CTA-4OHcinn, the propidium iodide stain (red color) targets membrane disruption and indicates membrane compromised cells (**i** and **j**, respectively).

The previous simulations showed that the CTA-4OHcinn micelle was not able to disrupt an asymmetric bilayer composed of Re-LPS and POPE, even after decreasing the LPS concentration from 75 to 25%. Next, we simulated a CTA-4OHcinn micelle above a modified asymmetric bilayer composed of 25% LPS and 75% POPG (System s27 from Table S-1). This time micelle merging occurred following adsorption, assimilation, and defect formation (Fig. 3a–d). Like the disruption of symmetric bilayers, cetrimonium was inserted continuously following the flip mechanism (Fig. 3e). The adsorbed sodium counterions also decreased because of the charge neutralization of the upper leaflet. This was confirmed by simulation snapshots that showed a considerable decrease in adsorbed sodium counterions after micelle merging (Fig. S-18c,d). The 4-OH cinnamate anions, carried in the micelle, were trapped within the bilayer, overcoming electrostatic repulsion (Fig. 3f). Based on the RDF analysis, it is likely that 4-OH cinnamate has two preferred configurations within the bilayer. It follows the orientation previously described for the trapped 4-OH cinnamate molecules in the symmetric bilayers, where the hydroxyl group is deeper inside the bilayer compared to the carboxylate. Additionally, it adopts a roughly parallel conformation to the bilayer surface with equidistant hydroxyl and carboxylate groups (Fig. 3g). The entrapment of 4OH cinnamate anions within the upper leaflet might also contribute to the biocidal effect of CTA-4OHcinn^56^. The lump defect mostly contains Re-LPS, consistent with the large volume that occupies these molecules undergoing a high lateral compression (Fig. 3h). The results demonstrate that POPG lipids promote faster bilayer disruption when the LPS proportion is below 25%. Additionally, confocal imaging targeting the bacterial wall of *K. pneumoniae* before and after 10 mM CTA-4OHcinn interaction was performed*.* The resultant images show that the inhibitor causes a widespread damage to the bacteria wall consistent with our simulations (Fig. 3i,j).

Thus far the mechanism by which an inhibitor micelle interacts with an outer and inner membrane of a gram-negative bacteria has been discussed. Between the outer and inner membrane, a thin peptidoglycan (PG) layer is also found. The peptidoglycan is a polymer mesh that gives structural support to the bacteria. The PG layer contains pores with sizes between 2–2.5 nm^64,65^. It is unlikely that whole inhibitor micelles will pass through the PG layer, since their average diameter is around 5 nm^26^. However, following the insertion mechanism documented here, it is likely that single cetrimonium and 4OH cinnamate ions will be able to pass through the PG pores and assemble above the inner membrane. Sharma and coworkers showed via all-atom molecular dynamics that the aggregation of dodecyl sulfate, myristate, palmitate, oleate, and stearate affected their pass through the PG. It was observed lower translocation times when the surfactants were at free monomer concentration^66^. This study could be extended to modelling the interaction between PG and cetrimonium 4OH-cinnamate in future work.

### Effect of CTA-4OHcinn on e-DNA

Along with targeting bacterial membranes, CTA-4OHcinn can inhibit biofilm formation on steel surfaces^14^. Since eDNA is one of the main components of biofilms^67^; it is hypothesized that CTA-4OHcinn can disrupt the normal functioning of eDNA, thus preventing biofilm formation. Therefore, the interaction between a cetrimonium/CTA-4OHcinn micelle and DNA was modelled (Systems s34 and s35 from Table S-1). All-atom simulations performed on adenine–thymine DNA (AT-DNA) and a cytosine–guanine DNA (CG-DNA) immersed in a solution containing 0.3 M CTA-4OHcinn showed that in both cases, spherical micelles self-assembled at the DNA-backbone (Fig. S-19a,b). It was proposed that micelle adsorption was driven by an electrostatic attraction between the cetrimonium amine groups and the DNA-backbone phosphates. Also, it is noticed that 4-OH cinnamate anions are stable within the micelle, keeping a close-proximity with the negative DNA-backbone and evidencing synergy (Fig. S-19c,d).

**Figure 4:**
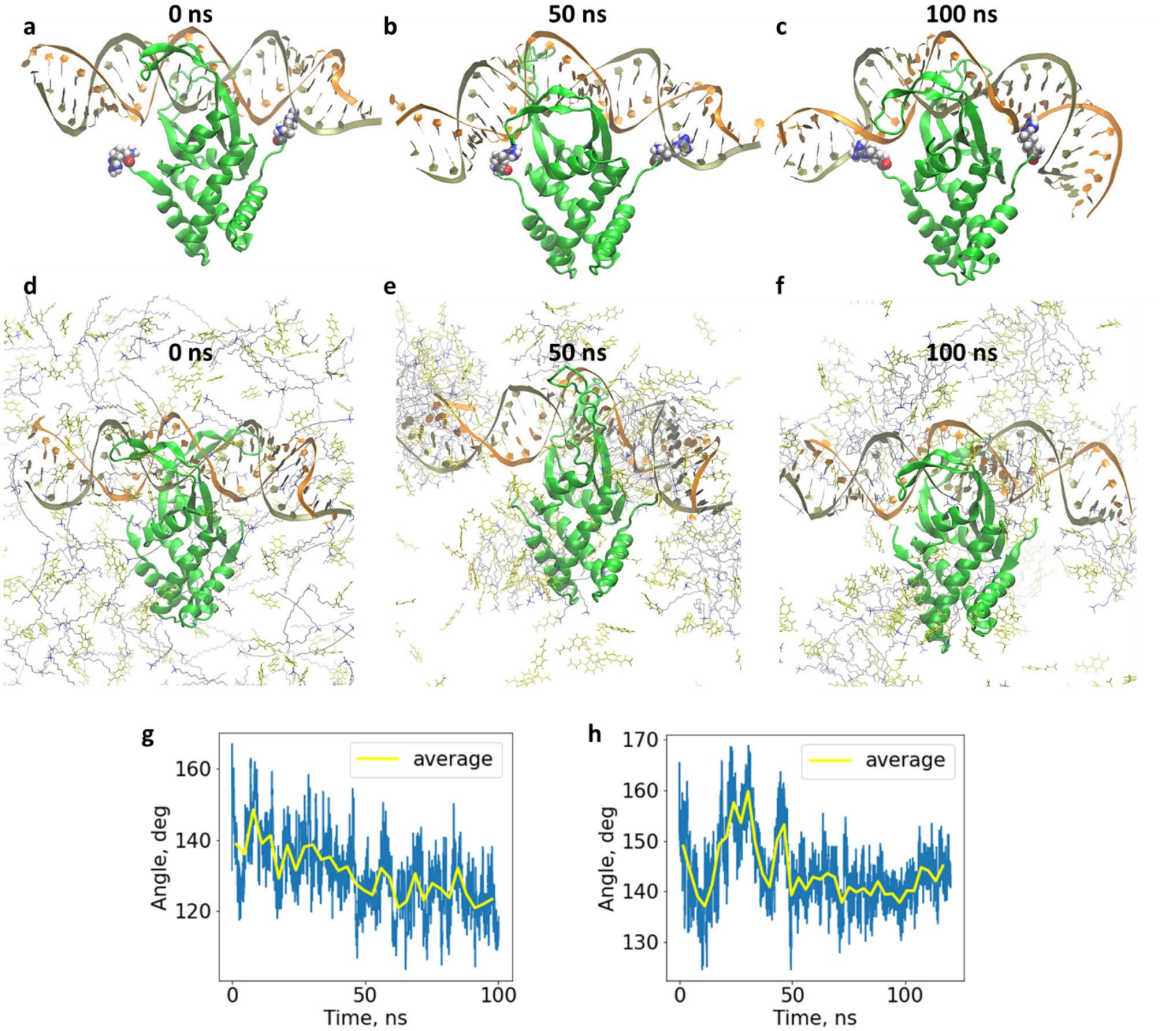
Simulation snapshots of a CG-DNA-Hbb complex at different timeframes, under normal conditions (**a**–**c**), and with CTA-4OHcinn (**d**–**f**). Bending degrees of DNA against time for the DNA-Hbb complex under normal conditions and with CTA-4OHcinn (**g** and **h**, respectively).

To test that the adsorbed micelles can hinder DNA packing, we proceed to model the Hbb-DNA complex (Systems s37 and s38 from Table S-1), where Hbb is a bacterial histone-like protein involved in DNA packing^46^. Simulations showed that under normal conditions Hbb can bend DNA via its terminal arginine residues that are electrostatically attracted to the DNA-backbone working as the “protein arms” (Fig. 4a–c). Additionally, DNA could be effectively coupled to Hbb because of the positive hydrogens located on the protein groove surface (Fig. S-10c). The results are consistent with the Hbb bending mechanism^30^. The DNA molecule reached a final bending angle of around 120° (Fig. 4g). When 0.2 M CTA-4OHcinn was present in the system (Systems s39 and s40 from Table S-1), the self-assembly of spherical micelles at the DNA-backbone and at the lower part of the protein was observed. The terminal arginine residues that were responsible for DNA bending, were weakly bonded to the DNA-backbone due to interference from the adsorbed micelles. Also the Hbb protein could not function properly (Fig. 4d–f) and the DNA molecule was not properly packed, as observed by the greater average bending angle of around 140° (Fig. 4h).

**Figure 5:**
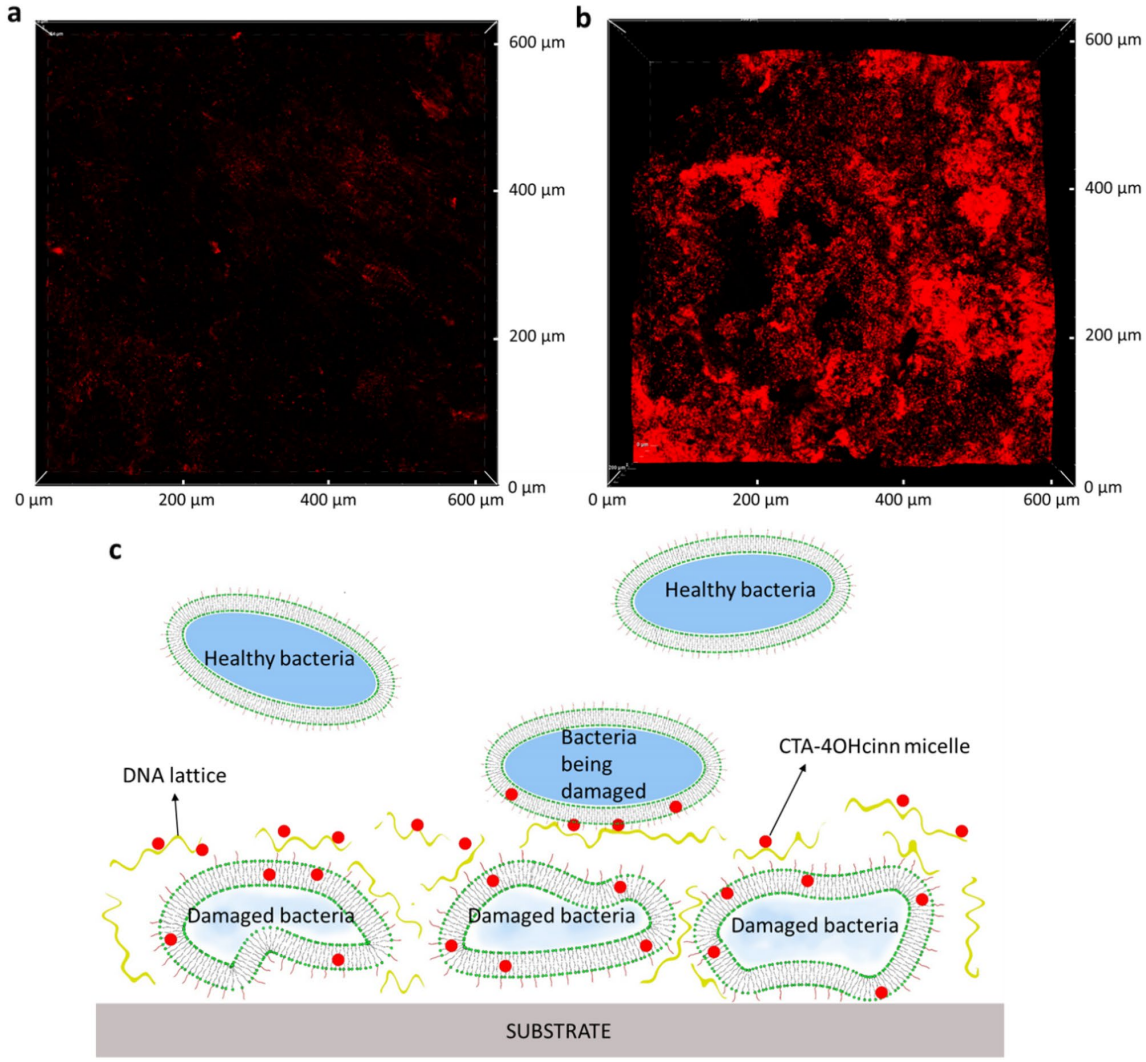
Confocal micrographs of a healthy biofilm (**a**) and a biofilm treated with 1 mM CTA-4OHcinn (**b**) on a mild steel surface; the red color represents extracellular DNA stained with propidium iodide. Proposed mechanism of biofilm inhibition by CTA-4OHcinn.

Via confocal analysis, it was observed that upon treatment of a *Pseudomonas balearica* biofilm with 1 mM CTA-4OHcinn a considerable amount of eDNA appears on a mild steel surface as a result of cellular lysis (Fig. 5a,b). This result, along with those from a recent study showing that CTA-4OHcinn can reduce both bacteria numbers and the surrounding EPS in a multi-species biofilm, confirm that the inhibitor is able to penetrate the mature biofilm^14^.

Simulations showed that the inhibitor micelles are adsorbed onto the DNA backbone, which could alter the interaction between DNA and histone-like proteins, as was demonstrated for the DNA-Hbb complex. Since DNA gives structural support to biofilms and it comprises a considerable proportion of the biofilm observed in MIC samples^14,50^, a defective biofilm will form, allowing the passage of more inhibitor, damaging the internal bacteria (Fig. 5c). The altered biofilm will also prevent further bacteria attachment, since the adsorbed micelles will form an antimicrobial coating with the DNA lattice, damaging any bacteria in close proximity; since simulations showed a strong electrostatic attraction between micelles and the DNA backbone. This hypothesis is consistent with the modelling and the previous experimental studies reporting the ability of CTA-4OHcinn to prevent the attachment of three bacterial strains (*Shewanella chilikensis*, *Pseudomonas balearica,* and *Klebsiella pneumoniae*) in a mature biofilm on an oxidized mild steel surface by around 99%^14^.

## Conclusions

This study demonstrates the potential of CTA-4OHcinn to target microbial cell membranes and extracellular DNA within biofilms; and provides the foundations for the rational design of target-specific anti-biofilm compounds. CTA-4OHcinn synthesis was originally motivated by abiotic and biotic corrosion studies on mild steel. However, this compound might have wider applications preventing biofilm formation on other surfaces such as medical devices and food processing environments, since its low toxicity against multicellular species was previously shown.

The mechanism by which CTA-4OHcinn hinders biofilm formation considers the interaction between the inhibitor and bacterial membranes as well as external DNA. Membrane disruption is observed between the micelle and a POPG symmetric bilayer, where the micelle is initially adsorbed to the bilayer surface via electrostatic attraction between the cetrimonium amine groups and the phospholipid phosphates. Cetrimonium molecules from the micelle are then injected within the bilayer’s upper leaflet following a flip mechanism, by which the adsorbed cetrimonium whose alkyl chain was initially pointing outwards from the bilayer is flipped inside due to Van der Waals interactions with the phospholipids alkyl chains. Eventually the micelle is integrated within the bilayer, creating a lump defect composed of a mixture of CTA-4OHcinn as well as some phospholipids. The micelle also acts as a carrier of 4OH-cinnamate anions, trapping them inside the bilayer once the defect is formed. When POPG is substituted by POPE, which is a neutral lipid, the micelle was not able to bond to the bilayer surface. Therefore, it is likely that electrostatic attraction is the main driving force for micelle attachment.

When the micelle was simulated with an asymmetric bilayer containing LPS and POPE, with varying proportions of POPE from 75 to 25%, micelle binding occurred on the bilayer surface due to electrostatic attraction between the cetrimonium amine groups and the negative oligosaccharides from LPS. 4OH-cinnamate anions were trapped in the bilayer upper leaflet overcoming electrostatic repulsion with the LPS oligosaccharides, demonstrating again the carrier role of the micelle.

In addition to targeting bacterial membranes, simulations show that CTA-4OHcinn forms micelles on the DNA backbone; due to electrostatic attraction between the cetrimonium amine groups and the DNA phosphates. Since it was observed experimentally that e-DNA is a significant component on biofilms; it is likely that this DNA lattice will be impregnated with inhibitor micelles. The modified e-DNA contains adsorbed inhibitor micelles that could attack the cellular membranes of approaching bacteria, preventing its proliferation on the surface.

CTA-OHcinn was also able to hinder DNA packaging with the histone-like protein hbb, by increasing the backbone rigidity. Such finding could be related with an additional inhibition mechanism by which e-DNA is not able to condense properly within the biofilm. The altered DNA network could lead to the formation of a more permeable biofilm, allowing the intrusion of more biocides which will reach inner bacteria.

While the mechanisms modelled here are consistent with experimental results that showed CTA-OHcinn was able to disrupt *K. pneumoniae* cell membranes and a mature, multi-species bio-film, there are always computational constraints that may be overcome in future works to improve our understanding. Most obviously here was the use of a rough LPS to model the bacteria outer membrane. Since a smooth LPS is composed of repeating units of saccharide units that extend several nanometers away; it will be harder for a CTA-4OHcinn micelle to penetrate such a bilayer. However, as our simulations demonstrate, there may be some threshold regarding the LPS proportion below which bilayer disruption might occur. Additionally, the CHARMM all-atom force field had to be used to describe the DNA–protein interactions and, therefore, simulation time was limited to 130 ns. Although the Martini CG model can model DNA and proteins, it uses bond constraints which do not allow the modelling of protein folding/unfolding and DNA bending events^33^. Nevertheless, despite these limitations, we clearly demonstrate here the potential to use computational techniques to target inhibitor and biofilm disruption for protecting against bacterial attack in applications as diverse as medical implants to water treatment and oil and gas exploration.

## Supplementary Information

Supplementary Information.

## Data Availability

The datasets used and/or analysed during the current study available from the corresponding author on reasonable request.
