# Supplementary material for: Effect of cetrimonium carrier micelles on bacterial membranes and extracellular DNA, an in silico study
Source: Sci Rep. 2023 May 17;13:8041. doi: 10.1038/s41598-023-32475-x (PMC10192197; doi:10.1038/s41598-023-32475-x)
Supplement: Supplementary file 1 — Supplementary Information. [file 41598_2023_32475_MOESM1_ESM.docx]

SUPPLEMENTARY INFORMATION

Simulation details

The membrane systems were simulated using the Polarizable Water Martini Coarse Grained (PW-Martini) model^1^. Following the proposed protocol an initial minimization was performed on the systems using a steep integrator and next the MD production was run using an NpT ensemble. A leapfrog integrator was used with a time step of 0.02 ps. Electrostatic interactions were calculated using a Reaction Field algorithm with a 1.1 nm cut-off, similar to the Van der Waals cut-off. Temperature coupling was performed using velocity rescaling^2^ keeping a constant value of 310 K. Regarding the pressure, independent couplings in the Z and XY dimensions were performed to avoid the buildup of tension in the bilayer. The pressure was set up to 1 bar and controlled by a Berendsen barostat^3^. Before the micelle-bilayer simulations, all the bilayers were stabilized for at least 10 µS. For modelling the micelles, a simulation box of 8x8x8 was used, within which 94 cetrimonium and 4OH cinnamate ions were randomly placed and the system was run for around 1 µs. The resulting micelle was extracted and placed above the corresponding bilayer.

Regarding DNA and protein simulations, these were performed using the CHARMM all atom forcefield. All the systems were pre-equilibrated with an NVT and then an NpT ensemble for at least 20 ns, prior to the MD production. An annealing process using the NVT ensemble is adopted to improve the dynamics, where the system was heated from 300 to 400 K and cooled from 400 to 300 K in the first 10 ns. The following 10 ns were run in an NpT ensemble at 300 K and 1 bar. The temperature and pressure were controlled using a Nose-Hoover thermostat and a Parrinello-Rahman barostat. For the bulk phase simulations, a final MD production was run with 100 ns at 300 K and 1 bar. The Van der Waals and electrostatic interactions had a cut-off distance of 1.4 nm and the PME method with a grid spacing of 0.16 was used to correct the electrostatic interaction across periodic simulation boxes. The leap frog integrator was used to calculate the total energy in each step size (2 fs) and the hydrogen-carbon bonds were constrained with the LINCS algorithm.

For modelling the DNA-protein systems, 16 and 32 base pairs were constructed using the NAFlex server. Subsequently, the DNA molecules were relaxed and simulated in water and counterions for 100 ns. The final DNA structures were extracted, and initial DNA-protein complexes were built with the aid of Python scripting. First, the coordinates of the proteins were extracted and converted to a numpy array. The proteins main axis was then aligned with the z-axis via a rotation and translation matrix. The coordinates of the DNA were then extracted and again via a rotation and translation matrix, the DNA was located within the protein pocket. The final coordinates were transformed into a Gromacs coordinate file with the *gro* extension. The system was solubilized with water molecules and neutralized with sodium or chloride counterions. For studying the interaction of the inhibitor with the DNA complex, cetrimonium and 4OH cinnamate ions were added randomly to the system substituting water molecules.

The different orientation of the DNA-protein complex observed in Fig. 4a and 4d is due to the minimization carried out with and without cetrimonium 4OH cinnamate. The presence of the inhibitor adds new interactions to the system which will alter the relaxed structure of the complex. Finally, a typical energy minimization routine was performed on the initial setups to correct overlapping atoms. Table S- 1 shows a summary of the performed simulations.

All RDF calculations were done with a bin width of 0.002 nm, which was the default value in Gromacs.

Table S- 1: Summary of the simulations

|  | **System composition** | **Box size, nm** | **Forcefield** |
| --- | --- | --- | --- |
| s1 | Octanol 962, water 1751, 4OH Cinn 1, Na 1 | 7x7x10 | PW-Martini |
| s2 | Octanol 962, water 5442, 4OH Cinn 1, sodium 1 | 6x6x10 | CHARMM |
| s3 | Octanol 962, water 5442, 4OH Cinn 1, sodium 1 | 6x6x10 | OPLS-AA |
| s4 | POPE 200, water 5942 (single bilayer) | 8x8x15 | PW-Martini |
| s5 | POPE 200, water 5732, Na 200 (single bilayer) | 8x8x15 | PW-Martini |
| s6 | POPG 84, POPE 116, water 5861, Na 84 (single bilayer) | 8x8x15 | PW-Martini |
| s7 | POPG 100, POPE 100, water 5838, Na 100 (single bilayer) | 8x8x15 | PW-Martini |
| s8 | POPG 120, POPE 80, water 5817, Na 120 (single bilayer) | 8x8x15 | PW-Martini |
| s9 | POPG 140, POPE 60, water 5801, Na 140 (single bilayer) | 8x8x15 | PW-Martini |
| s10 | POPE 400, CET 94, water 10590, Cl 94 (double bilayer) | 8x8x28 | PW-Martini |
| s11 | POPG 400, CET 94, water 10302, CL 94, Na 400 (double bilayer) | 8x8x26 | PW-Martini |
| s12 | POPG 168, POPE 232, CET 94, water 10382, Cl 94, Na 168 (double bilayer) | 8x8x27 | PW-Martini |
| s13 | POPE 400, 4OH-Cinn 94, water 10584, Na 94 (double bilayer) | 8x8x27 | PW-Martini |
| s14 | POPG 400, 4OH-Cinn 94, water 10481, Na 494 (double bilayer) | 8x8x27 | PW-Martini |
| s15 | POPG 168, POPE 232, 4OH-Cinn 94, water 10376, Na 262 (double bilayer) | 8x8x27 | PW-Martini |
| s16 | POPE 400, CET 94, 4OH-Cinn 94, water (double bilayer) | 8x8x27 | PW-Martini |
| s17 | POPG 400, CET 94, 4OH-Cinn 94, water (double bilayer) | 8x8x26 | PW-Martini |
| s18 | POPG 168, POPE 232, CET 94, 4OH-Cinn 94, water 10382, Na 168 (double bilayer) | 8x8x27 | PW-Martini |
| s19 | POPG 200, POPE 200, CET 94, 4OH-Cinn 94, water 10478, Na 200 (double bilayer) | 8x8x27 | PW-Martini |
| s20 | POPG 200, POPE 200, 4OH-Cinn 94, water 10252, Na 294 (double bilayer) | 8x8x27 | PW-Martini |
| s21 | POPG 280, POPE 120, CET 94, 4OH-Cinn 94, water 10294, Na 270 (double bilayer) | 8x8x27 | PW-Martini |
| s22 | LPS 113, POPE 84, CET 94, water 11839, Na 504, Cl 94 (double bilayer) | 9x9x27 | PW-Martini |
| s23 | LPS 113, POPE 84, CET 94, 4OH-Cinn 94, water 11839, Na 504, Cl 94 (double bilayer) | 9x9x27 | PW-Martini |
| s24 | LPS 30, POPE 141, water 2497, Na 180 (single bilayer) | 8x8x10 | PW-Martini |
| s25 | LPS 171, POPE 171, CET 94, 4OH-Cinn 94, water 12087, Na 380 (double bilayer) | 9x9x29 | PW-Martini |
| s26 | LPS 10, POPG 162, water 2068, Na 276 (single bilayer) | 8x8x11 | PW-Martini |
| s27 | LPS 38, POPE 324, CET 94, 4OH-Cinn 94, water 11023, Na 3552 (double bilayer) | 9x9x28 | PW-Martini |
| s28 | LPS 91, POPG 273, POPE 200, water 62003, Na 819 (Vesicle) | 21x21x21 | PW-Martini |
| s29 | LPS 91, POPG 273, POPE 200, CET 386, 4OH-Cinn 386, water 61017, Na 819 (Vesicle) | 21x21x21 | PW-Martini |
| s30 | AT-DNA 1, water 15187, Na 32 | 8x8x8 | CHARMM |
| s31 | CG-DNA 1, water 14945, Na 32 | 8x8x8 | CHARMM |
| s32 | AT-DNA 1, CET 80, water 12863, Na 32, Cl 80 | 8x8x8 | CHARMM |
| s33 | CG-DNA 1, CET 80, water 13060, Na 32, Cl 80 | 8x8x8 | CHARMM |
| s34 | AT-DNA 1, CET 80, 4OH-Cinn 80, water 12530, Na 32 | 8x8x8 | CHARMM |
| s35 | CG-DNA 1, CET 80, 4OH-Cinn 80, water 12905, Na 32 | 8x8x8 | CHARMM |
| s36 | Hbb 1, water 16311, Cl 20 | 8x8x8 | CHARMM |
| s37 | Hbb1, AT-DNA 1, water 47135, Na 40 | 12x12x12 | CHARMM |
| s38 | Hbb1, CG-DNA 1, water 47135, Na 40 | 12x12x12 | CHARMM |
| s39 | Hbb1, AT-DNA 1, CET 200, 4OH-Cinn 200, water 48238, Na 40 | 12x12x12 | CHARMM |
| s40 | Hbb1, CG-DNA 1, CET 200, 4OH-Cinn 200, water 47995, Na 40 | 12x12x12 | CHARMM |

Figure S- 1 shows the molecular structure of a POPE and a POPG lipid with their respective coarse-grained models, extracted from Martini lipidome^4^. Notice that POPE has a neutral charge while in the case of POPG, it has a negative charge of -1.


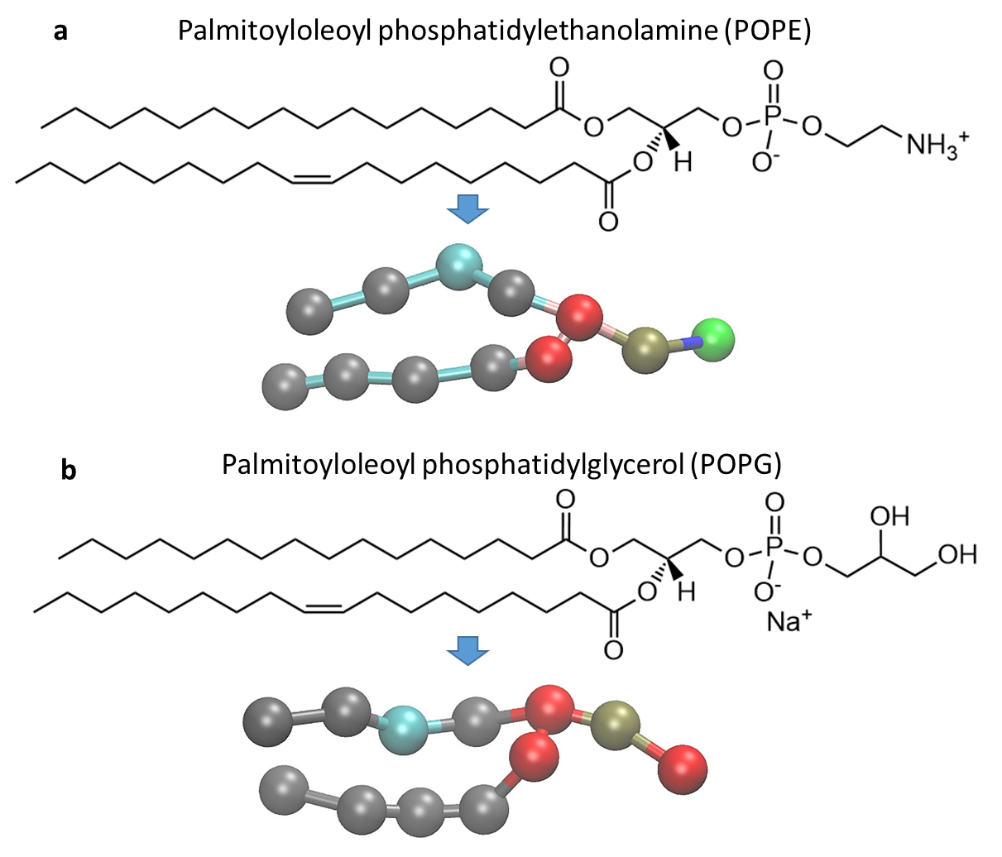


Figure S- 1: Molecular structure of POPE and POPG phospholipids with their respective coarse-grained models (a and b, respectively).

Figure S- 2 shows the molecular structure of a Re-LPS from *Escherichia coli* and its respective coarse-grained representation^5^.


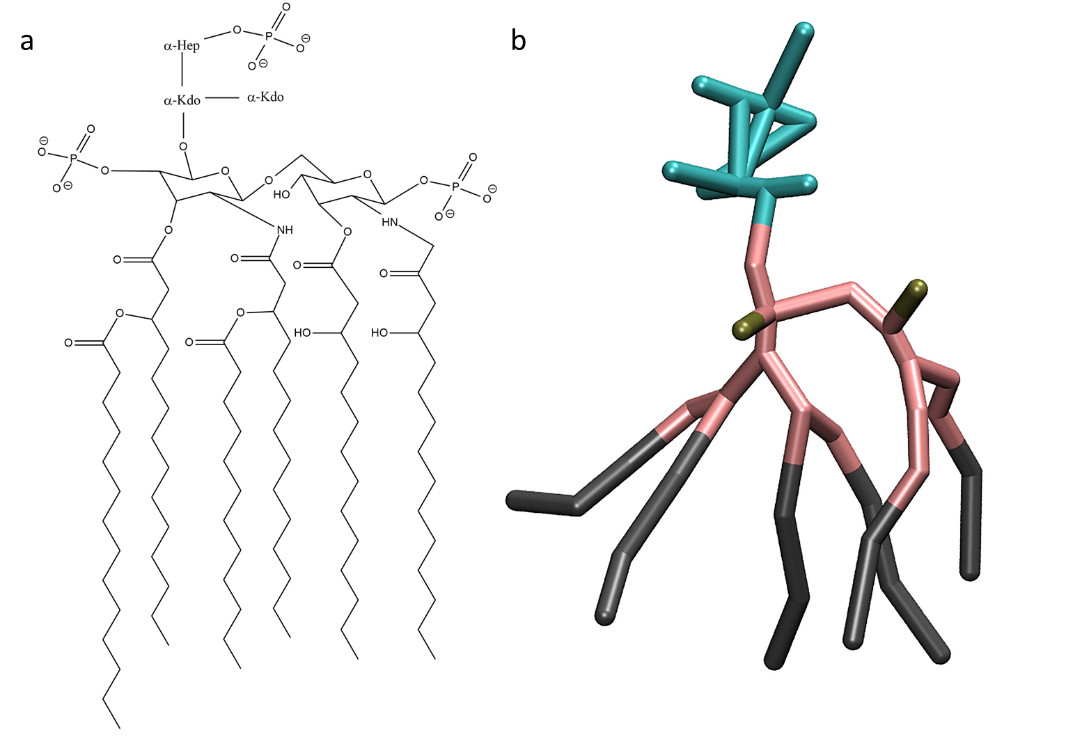


Figure S- 2: Molecular structure of a Re-LPS from Escherichia coli (a) and its coarse grained representation (b).

Figure S- 3 a, b and c shows simulation snapshots of POPE, POPG and 58%POPE + 42%POPG bilayers, respectively. These bilayers were stable within the simulation time, around 12 µs. The predicted bilayer thickness was extracted from the phosphate density profile plots (Figure S- 3 d, e and f), resulting in 3.9 nm, 3.86 nm and 3.83 nm for POPE, POPG and 58%POPE + 42%POPG, correspondingly. The experimental thickness of POPE and POPG bilayers are 3.75 nm and 3.85 nm, respectively^6^, which shows consistency with our models. Also, we calculated the area per lipid (APL) resulting in average values of 0.64 nm^2^, 0.68 nm^2^ and 0.66 nm^2^ for POPE, POPG and 58%POPE + 42%POPG, correspondingly (Figure S- 3 g, h and i). Such values are consistent with their experimental counterpart that reports APL’s of 0.59 nm^2^ and 0.67 nm^2^ for POPE and POPG bilayers, respectively^6^.


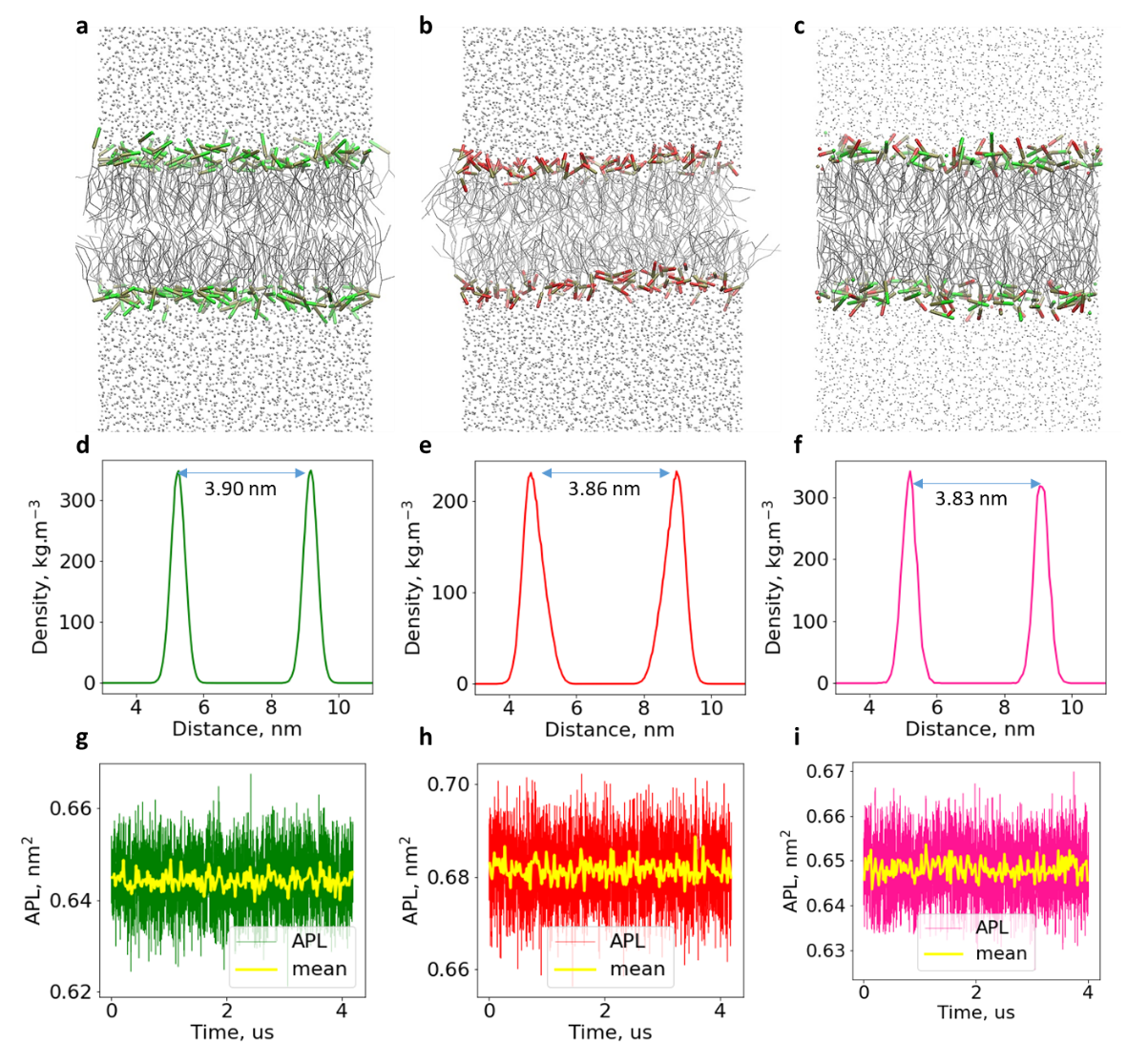


Figure S- 3: Simulation snapshots of a POPE, POPG and 58% POPE + 42% POPG bilayer (a, b and c, respectively). Phosphate density profile in the normal direction of the bilayer for POPE, POPG and 58% POPE + 42% POPG bilayer (d, e and f, respectively). Area per lipid (APL) calculated in the last 4 µs for POPE, POPG and 58% POPE + 42% POPG bilayer (g, h and I, respectively).

Figure S- 4 shows the initial setup used for modelling micelle-bilayer interactions, the system dimensions might vary due to equilibration.


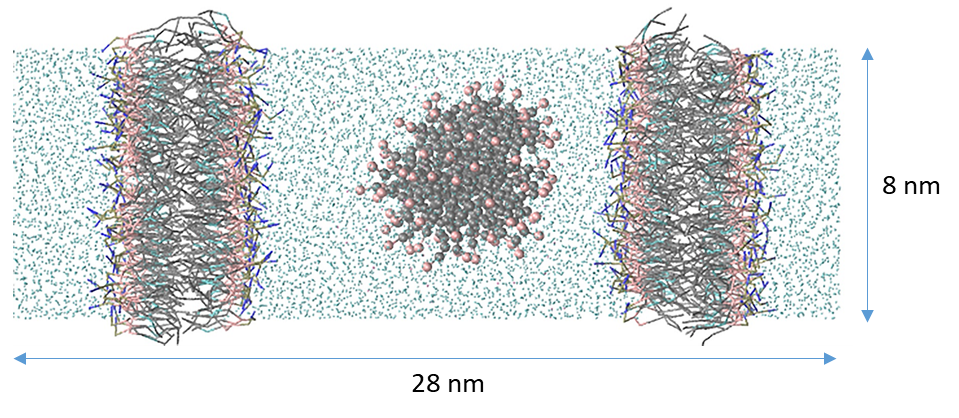


Figure S- 4: Initial setup for modelling micelle bilayer interaction, the dimensions might vary due to system equilibration.

Figure S- 5a shows the simplification of the cetrimonium all-atom to coarse grained model. Simulations are performed for a system containing 240 cetrimonium, 240 chloride counterions and 12000 water molecules, using the OPLS-AA and CHARMM all-atom forcefields, and the PW-Martini coarse-grained forcefield. The radial distribution functions of the amine group, chloride counterions and water, taking as a reference the terminal carbon (Figure S- 5 b, c and d, respectively), show that the PW-Martini model describes consistently the molecular interactions of cetrimonium. Final simulation snapshots of the system using the CHARMM and PW-Martini forcefields, further validate the cetrimonium coarse-grained model, by showing similar results (Figure S- 5 e and f). The simulations are consistent with experimental data^7^, showing that cetrimonium in aqueous solutions forms spherical micelles with a hydrophobic core, predicting a micelle radius of around 2.6 nm, which is in agreement with experimental data^8,9^.


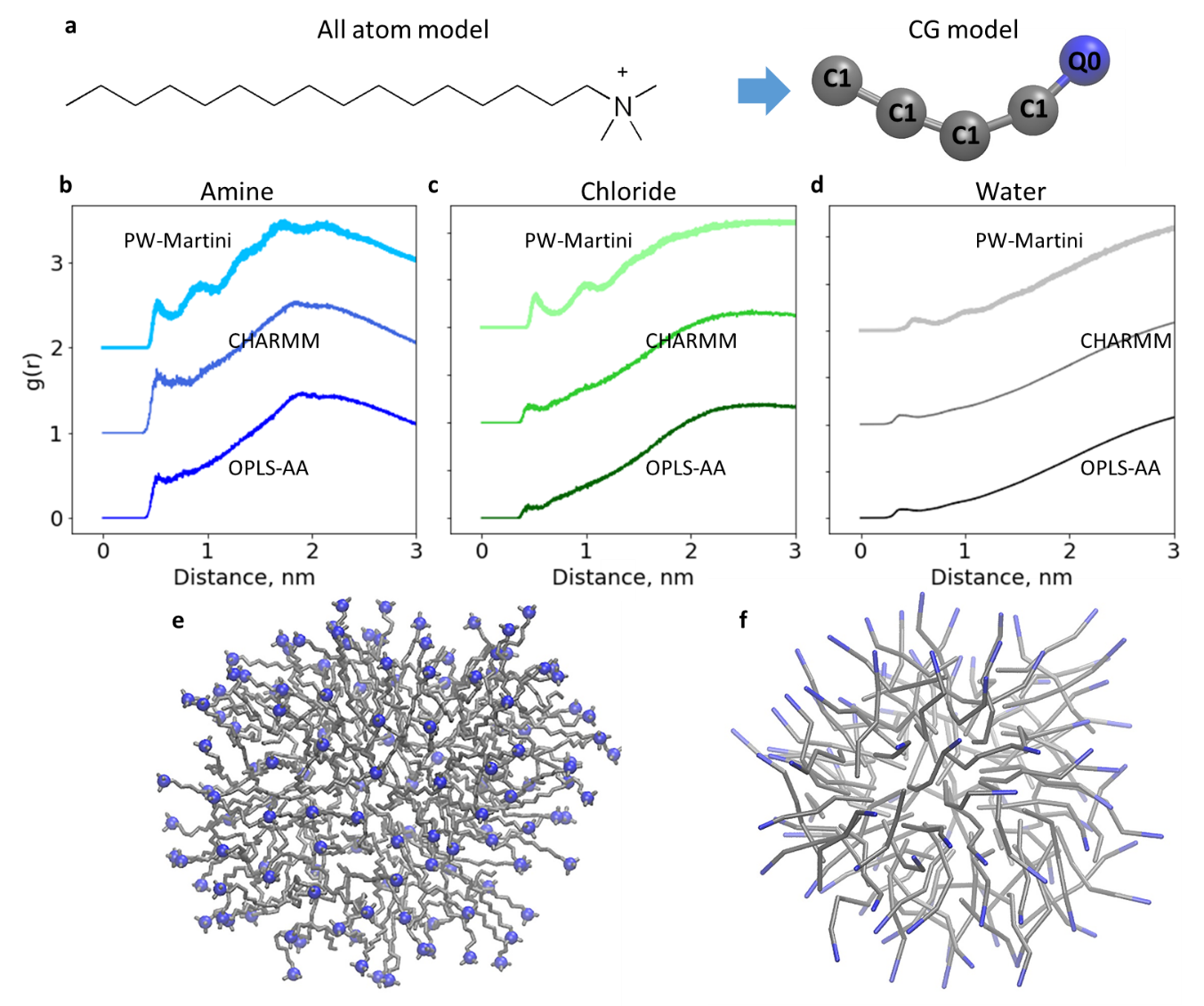


Figure S- 5: All atom and coarse-grained model of cetrimonium (a). The radial distribution functions of the amine group, chloride counterions and water, taking as a reference the terminal carbon. The CHARMM and PW-Martini curves were shifted upward by 1 and 2 units respectively (b, c and d, respectively). Simulation snapshots after 150 ns of a cetrimonium bromide using the CHARMM forcefield (e) and the PW-Martini model (f).

The construction of the 4-OH cinnamate CG model followed a bottom-up approach, i.e. beginning from its AA model. Figure S- 6a shows the overlap between martini coarse-grained beads and the atoms of 4-OH cinnamate. The free energy profiles for 4-OH cinnamate (Systems s1, s2 and s3, respectively from Table S- 1) migrating from water to an octanol phase were obtained using the OPLS-AA, CHARMM and PW-Martini forcefields (Figure S- 6b). The similarity between the profiles indicates that the CG model describes adequately the non-bonded interactions of 4-OH cinnamate. The resulting partition energies are 14.41 kJ/mol, 16.45 kJ/mol and 18.18 kJ/mol for the OPLS-AA, CHARMM and PW-Martini forcefields, respectively. Additionally, bond, angle and dihedral distributions were compared between CHRAMM and PW-Martini models (Figure S- 6 from c to i), indicating that the CG model also replicates bonded interactions.


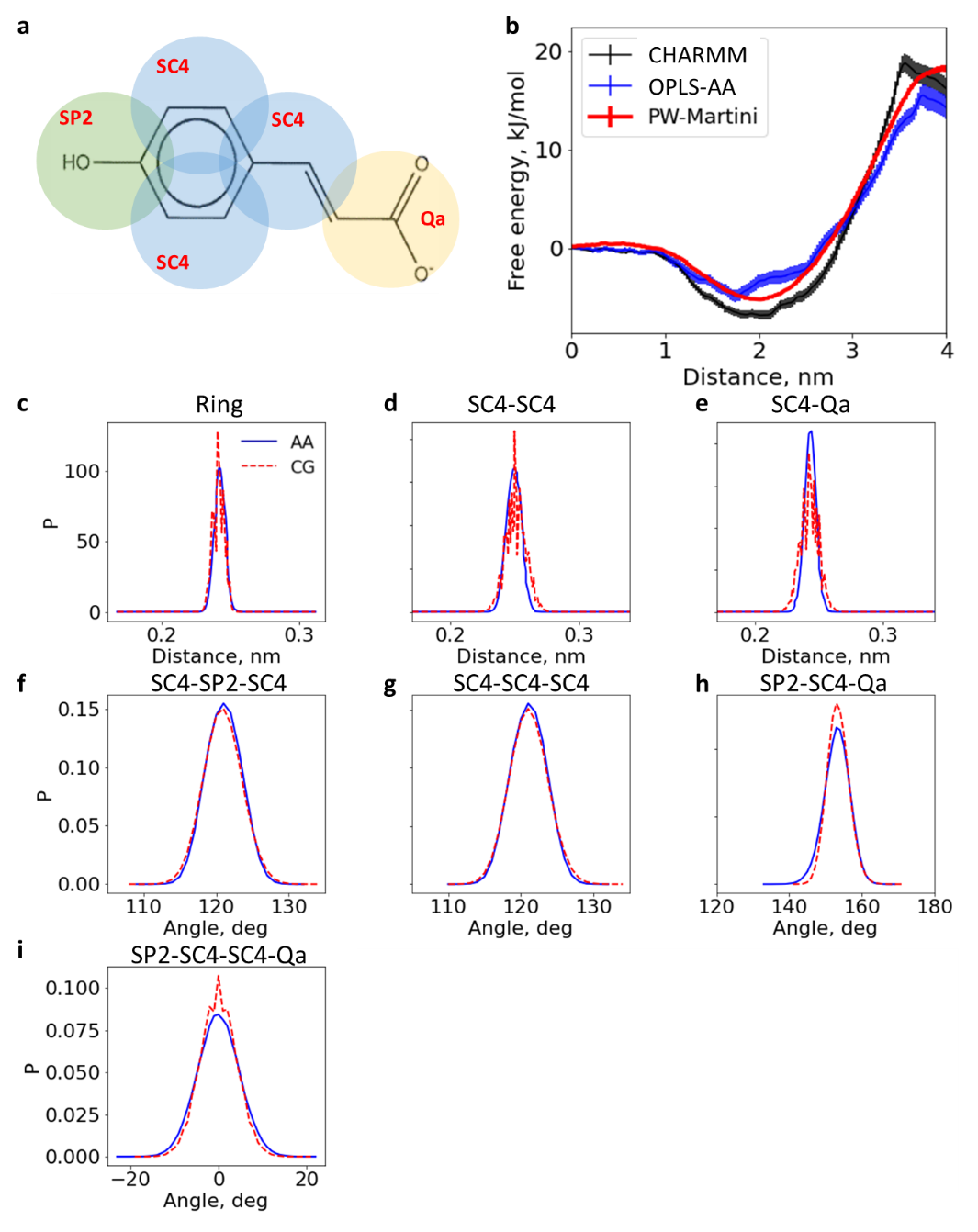


Figure S- 6: PW-Martini parameters for 4-OH cinnamate (a). Free energy profile of a 4-OH cinnamate molecule migrating from a water to an octanol phase calculated from simulations using CHARMM, OPLS-AA and PW-Martini forcefields (b). Bond length distribution for all-atom and CG model of 4-OH cinnamate (c, d and e). Angle distribution for all-atom and CG model of 4-OH cinnamate (f, g, h). Dihedral distribution for all-atom and CG model of 4-OH cinnamate (i).

The CG model for CTA-4OHcinn was further validated by comparing simulation results of the organic ions in water using the CHARMM and PW-Martini forcefields (Figure S- 7 a and b). In both cases, composed micelles are obtained where the aromatic anions are integrated within the cetrimonium micelles. We reported a similar ellipsoidal morphology in our previous study consistent with SAXS experiments^10^. Additionally, radial distribution functions of amine, carboxylate and hydroxyl groups were calculated taking as a reference the terminal carbon from the cetrimonium alkyl chain for the using the CHARMM and PW-Martini forcefields (Figure S- 7 c, d and e). Both distributions are similar, showing that the CG model captures the main interactions observed in the all-atom model.


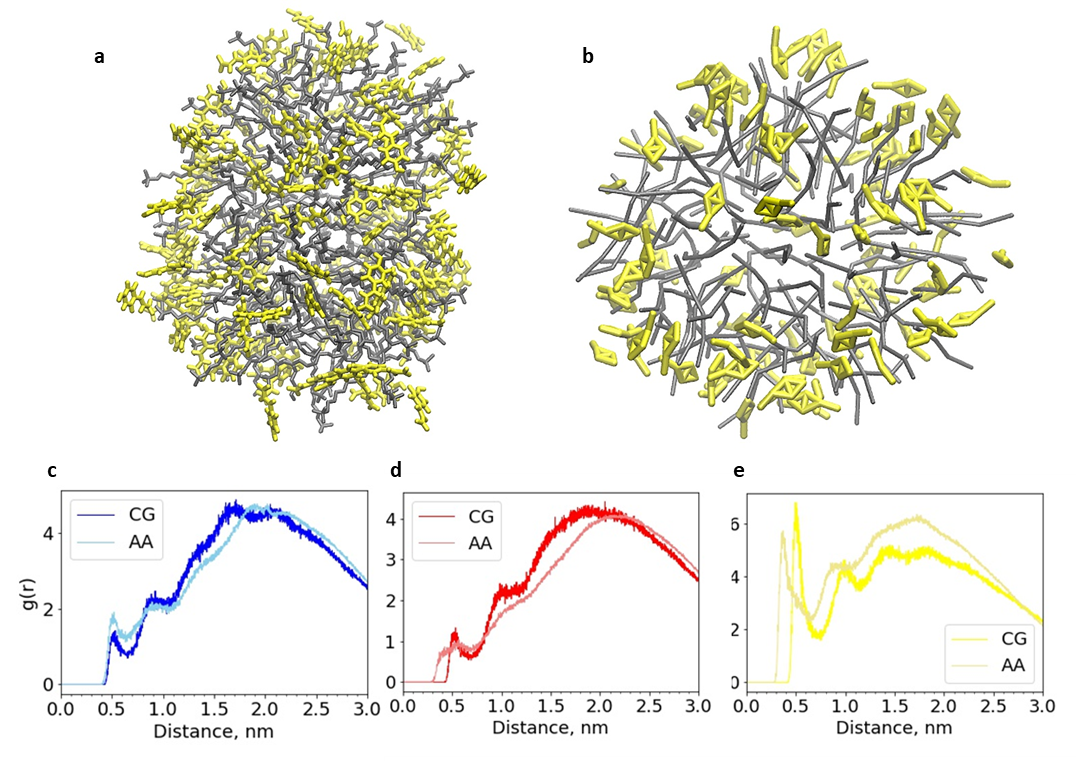


Figure S- 7: AA simulation of CTA-4OHcinn using the CHARMM and PW-Martini forcefields (a and b respectively). Radial distribution functions for amine, carboxylate and hydroxyl group taking as a reference the terminal carbon from cetrimonium alkyl chain for the CHARMM (AA) and PW-Martini (CG) forcefields (c, d and f, respectively).

Figure S- 8 a and b show the simulation snapshots of DNA molecules composed of 16 adenine–thymine and cytosine–guanine base pairs. It is noticed that in both systems, the DNA keeps its characteristic helical morphology. No constraints were used for the MD production and the simulations were performed using the all-atom CHARMM forcefield. Based on the RDF calculated between the backbone phosphates, the predicted diameter for AT-DNA and CG-DNA was 2.01 and 1.83 nm respectively (Figure S- 8 c and d); which is consistent with the experimental DNA diameter that varies between 1.8 and 2.3 nm^11^.


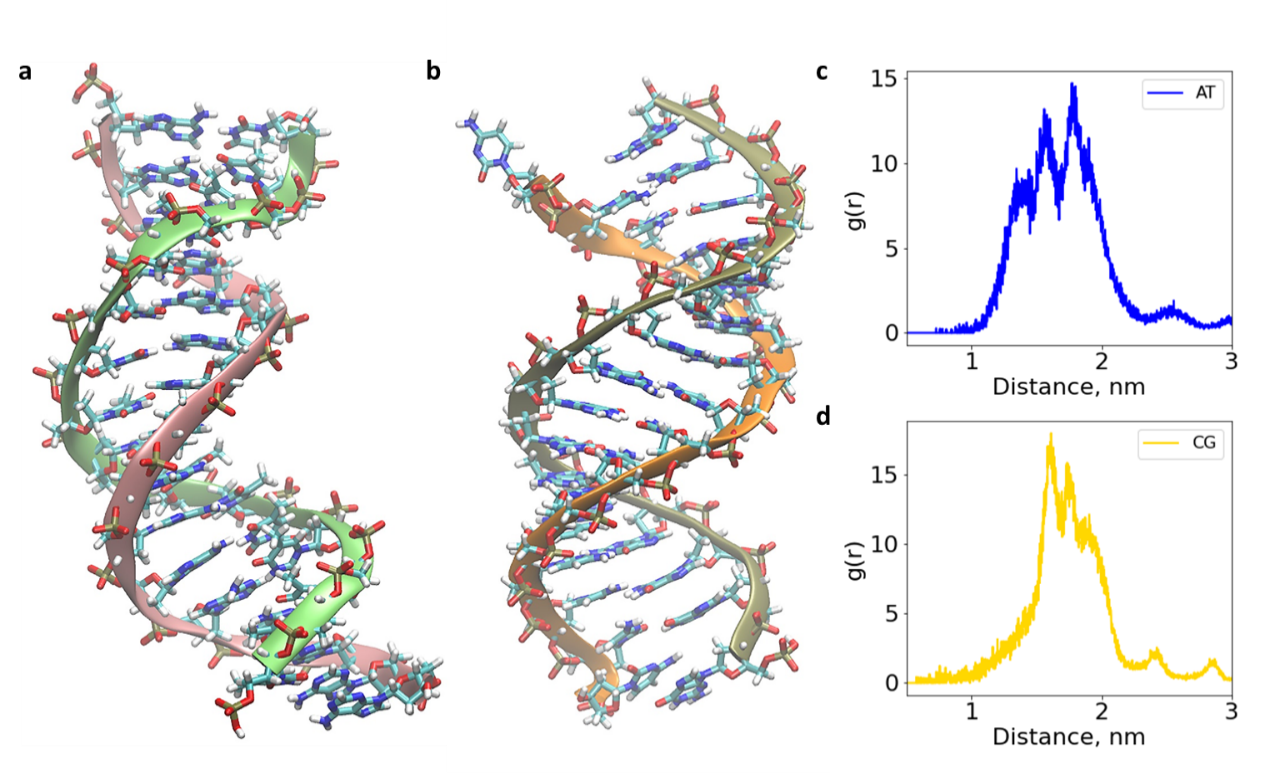


Figure S- 8: 100 ns simulation snapshots of DNA composed of 16 adenine–thymine and cytosine–guanine base pairs (a and b, respectively). RDF calculated between the phosphate backbones for both AT-DNA and CG-DNA sequences (c and d respectively).

The RDF calculated between the A-T and C-G pairs (Figure S- 9 a and b) shows that the DNA strands stabilizes by forming 2 and 3 hydrogen bonds, respectively, with a length less than 2 Å, which is consistent with literature^12^.


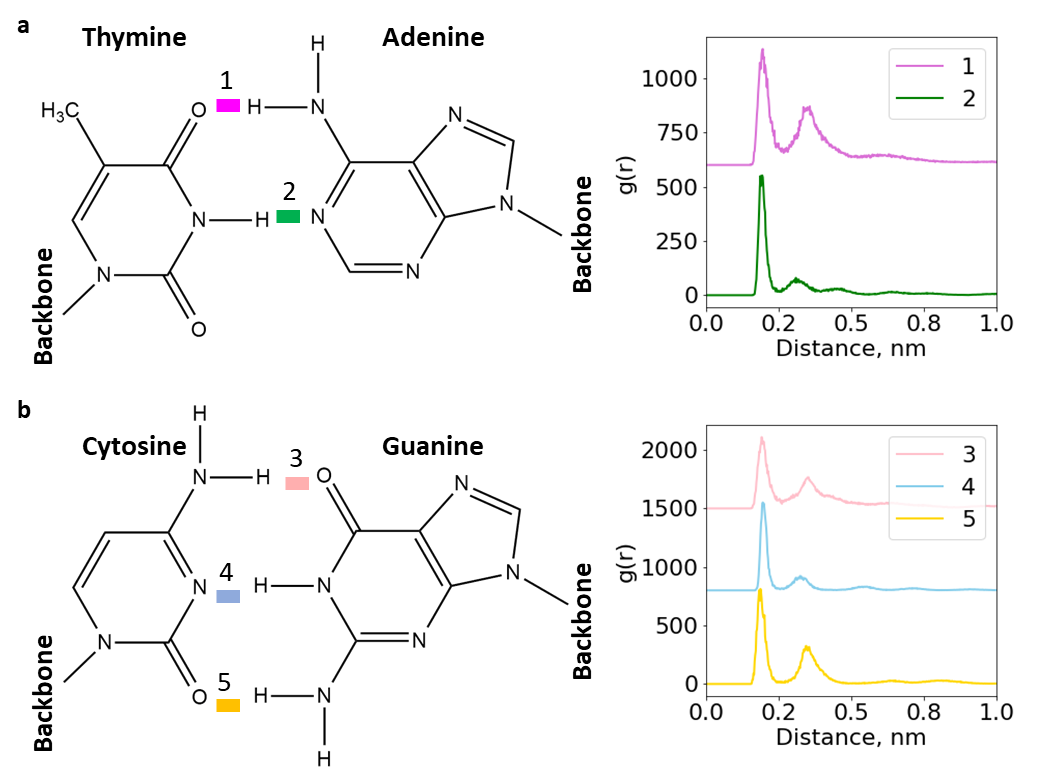


Figure S- 9: RDF calculated between O-H and H-N atoms from the thymine-adenine pair. The O-H curve was shifted upward 600 units (a) and between the O-H, H-N, and H-O atoms from the cytosine-guanine pair. The H-N and H-O curves were shifted upward 800 and 1500 units, respectively (b).

Hbb is a homodimer composed of two identical polypeptides as is observed in Figure S- 10 a and b. The loops that are found in the upper section of the structure act as molecular tweezers that trap DNA^13^. It was noticed that the groove formed by the two chains contains a considerable quantity of amine groups with positive hydrogen atoms on the surface (Figure S- 10c). Also, hydrogen bonds are formed between the chains, stabilizing the protein (Figure S- 10 d). The system is stable throughout the simulation time since its energy keeps a constant average value of around -579 MJ/mol (Figure S- 10 e).


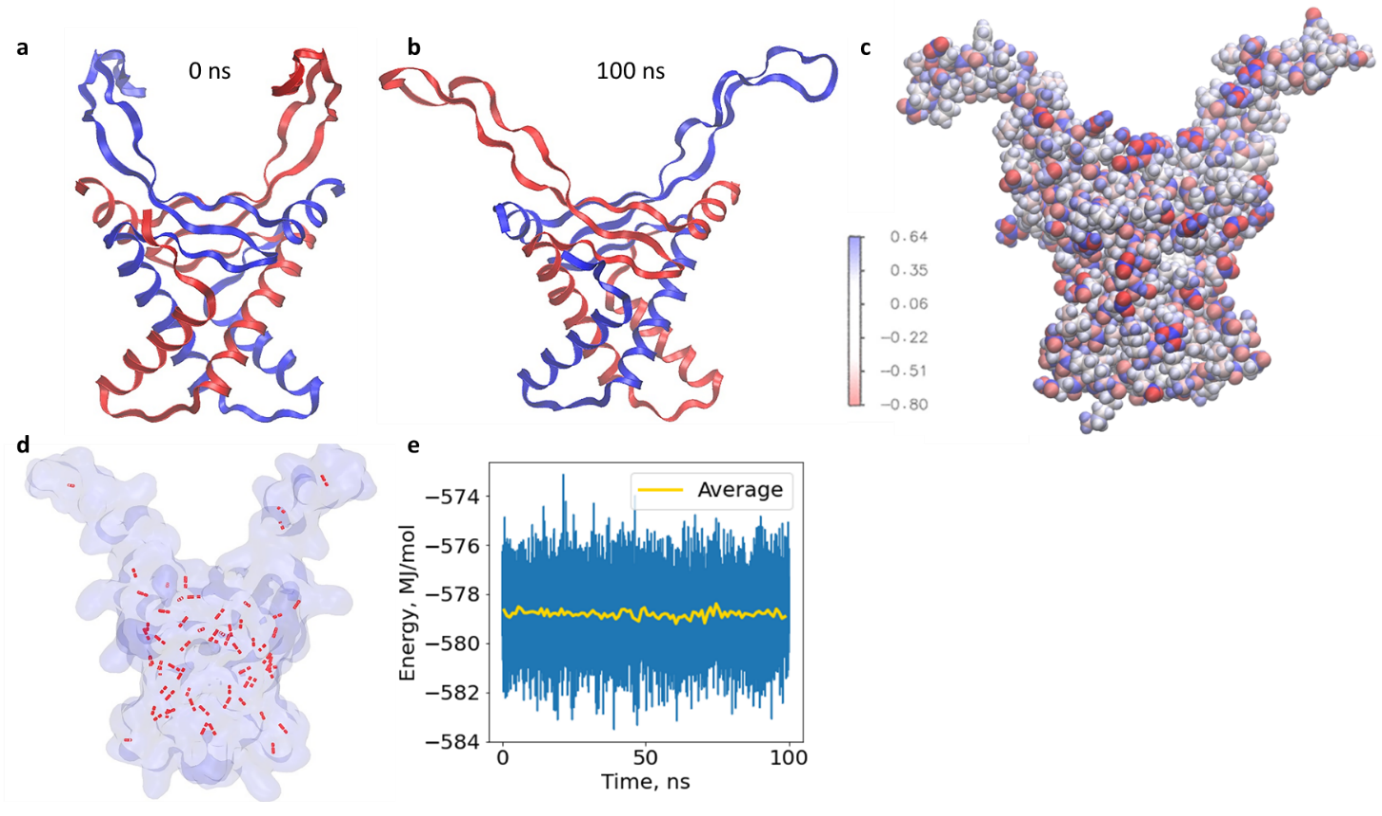


Figure S- 10: Simulation snapshots of Hbb protein, the two polypeptide chains are colored red and blue (a and b, respectively). Charge distribution of Hbb, where the red and blue colors represent negative and positive spots, respectively (c). Hydrogen bonds within the protein, colored in red (d). Energy evolution of the Hbb system (e).

Figure S- 11a shows that the cetrimonium micelle has little interaction with the POPE bilayer even after 30 µs. Notice that the bilayer surface is shielded by positive amino groups (green beads). RDF calculated for the amine, chloride and water taking as a reference the terminal carbon from the cetrimonium alkyl chain shows that the micelle keeps its integrity, with the amine polar heads located in the outer shell and the absence of water in the micelle core (Figure S- 11c). It is also noticed that the bilayer thickness is not altered (Figure S- 11b).


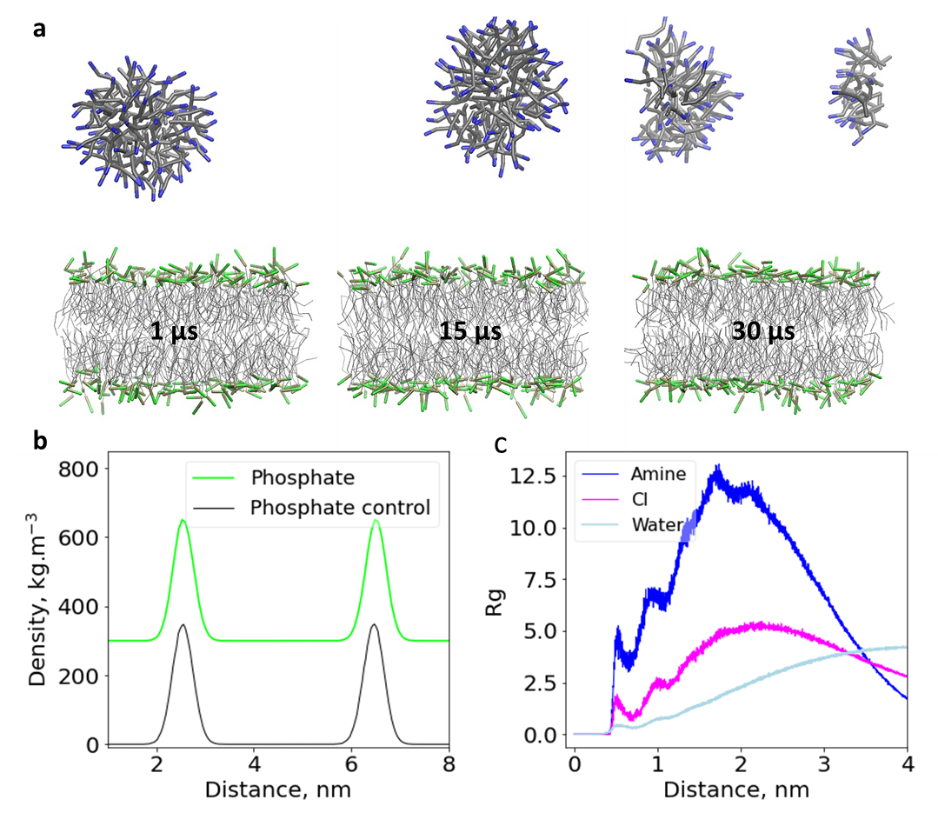


Figure S- 11: Simulation snapshots at different time frames of a cetrimonium micelle with chloride counterions and a POPE bilayer (a). Phosphate density profile of a POPE bilayer with and without a cetrimonium micelle (b). Radial distribution function of the amine group, chloride counterions and water, taking as a reference the terminal carbon from the cetrimonium alkyl chain (c).

Figure S- 12a shows the location of chloride counterions after micelle disruption regarding the interaction between a cetrimonium micelle and a bilayer composed of 52% POPE + 48% POPG. Figure S- 12b shows the density profile of chloride counterions along the bilayer normal direction.


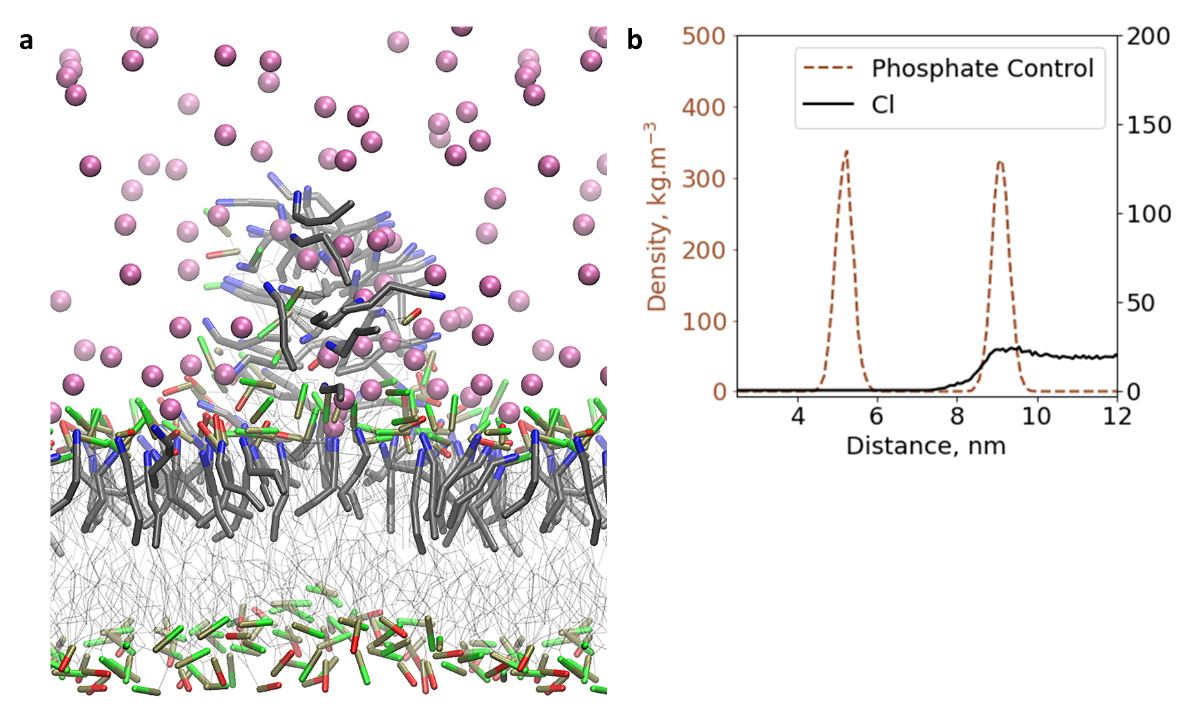


Figure S- 12: Simulation snapshots at 28 µs regarding the interaction between a cetrimonium micelle with chloride counterions (purple spheres) and a bilayer composed of 52% POPE + 48% POPG (a). Density profile of chloride counterions (b).

Figure S- 13 a-d shows the interaction of a CTA-4OHcinn micelle with a mixed bilayer composed of 52% POPE + 48% POPG. Notice that no micelle adsorption occurred in the first 15 µs. Figure S- 13e shows the flip mechanism by which cetrimonium inserts within the bilayer.


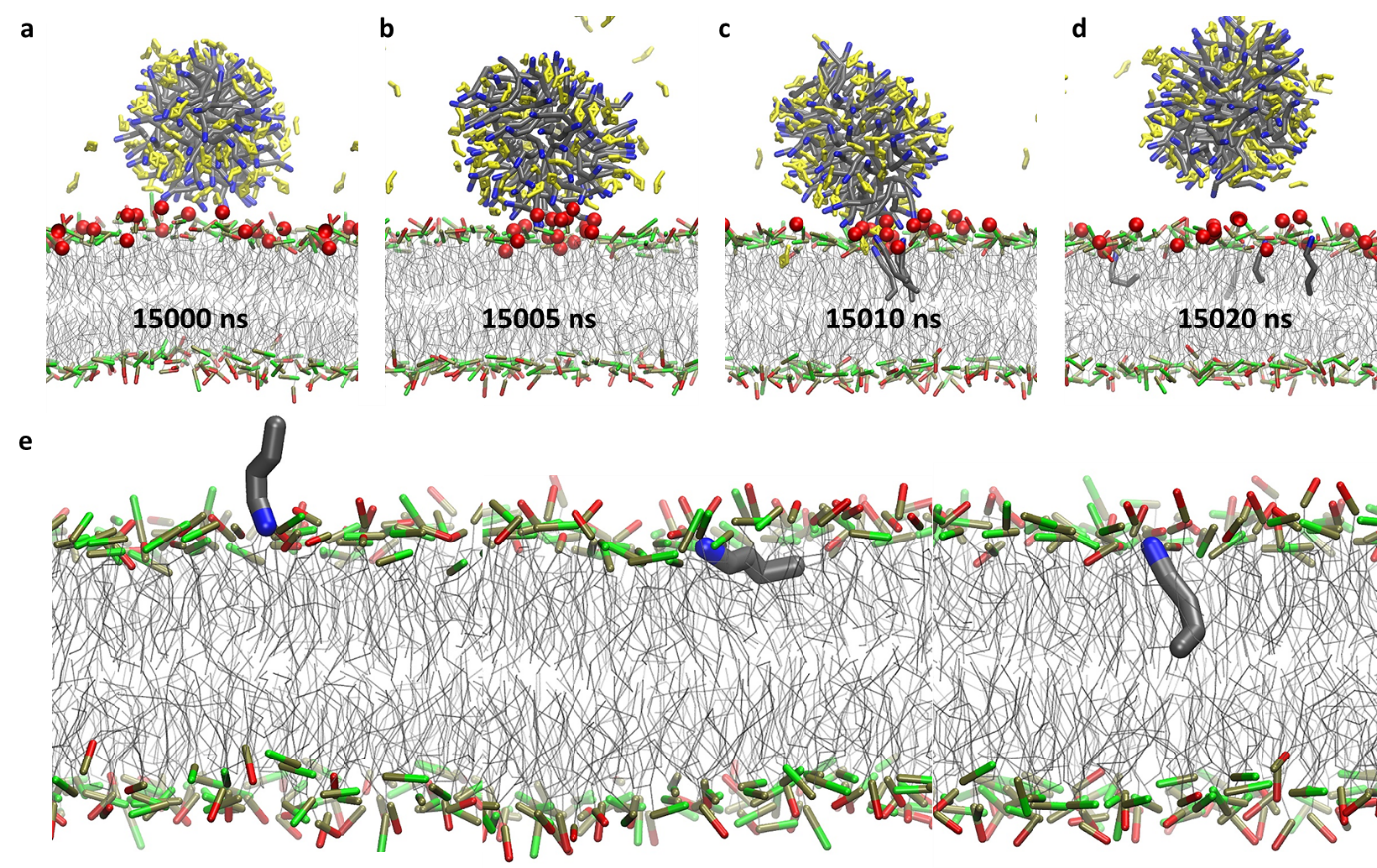


Figure S- 13: Interaction of a CTA-4OHcinn micelle with a bilayer composed of 52% POPE + 48% POPG; the red spheres represent the segregated POPG amine heads, POPE and POPG polar heads are colored green and red, respectively (a-d). The flip mechanism by which cetrimonium is inserted in the bilayer (e).

Figure S- 14a shows the segregation of POPG amine heads due to micelle adsorption and detachment from a 50% POPE + 50% POPG bilayer. Figure S- 14b shows the insertion mechanism of a cetrimonium molecule in a 50% POPE + 50% POPG bilayer.


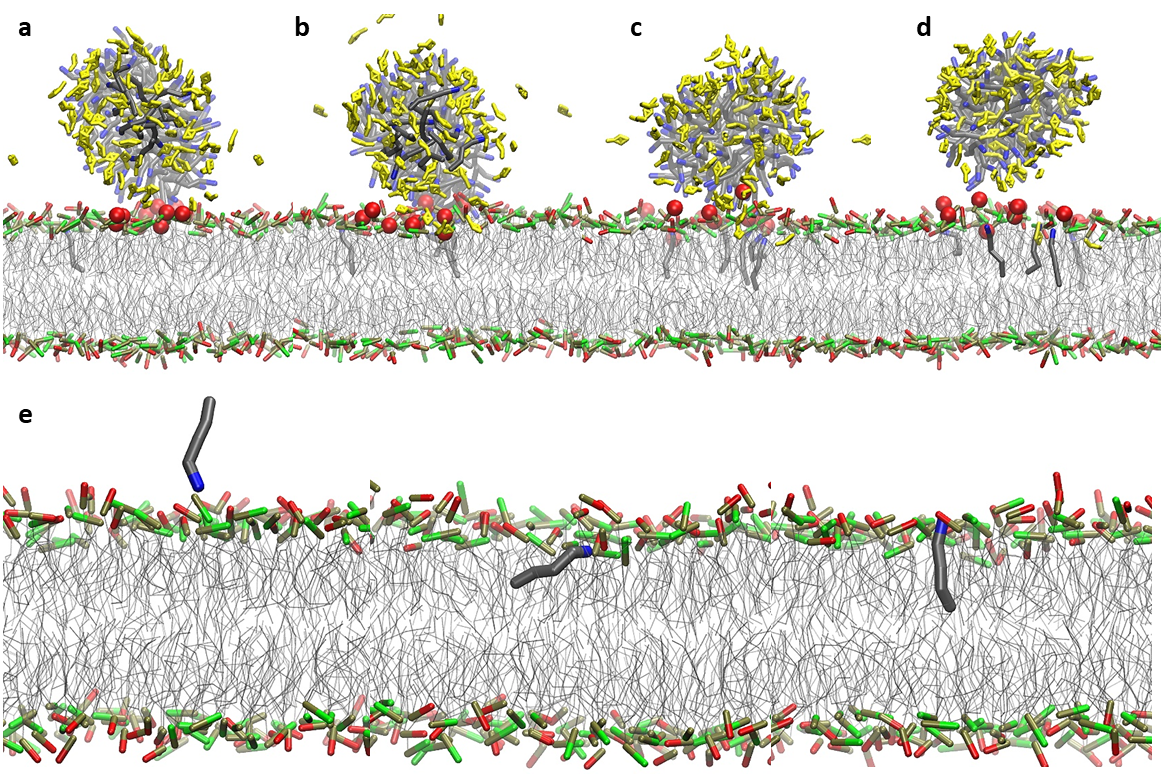


*Figure S- 14: Segregation of POPG polar heads as a result of micelle adsorption and detachment from a 50% POPE + 50% POPG bilayer; POPE and POPG polar heads are colored green and red, respectively (a-d). The flip mechanism for the insertion of a cetrimonium molecule in a 50% POPE + 50% POPG (e).*

Figure S- 15 shows the segregation of POPG lipids when a cetrimonium micelle and a CTA 4-OHcinn micelle is bonded to a mixed bilayer surface.


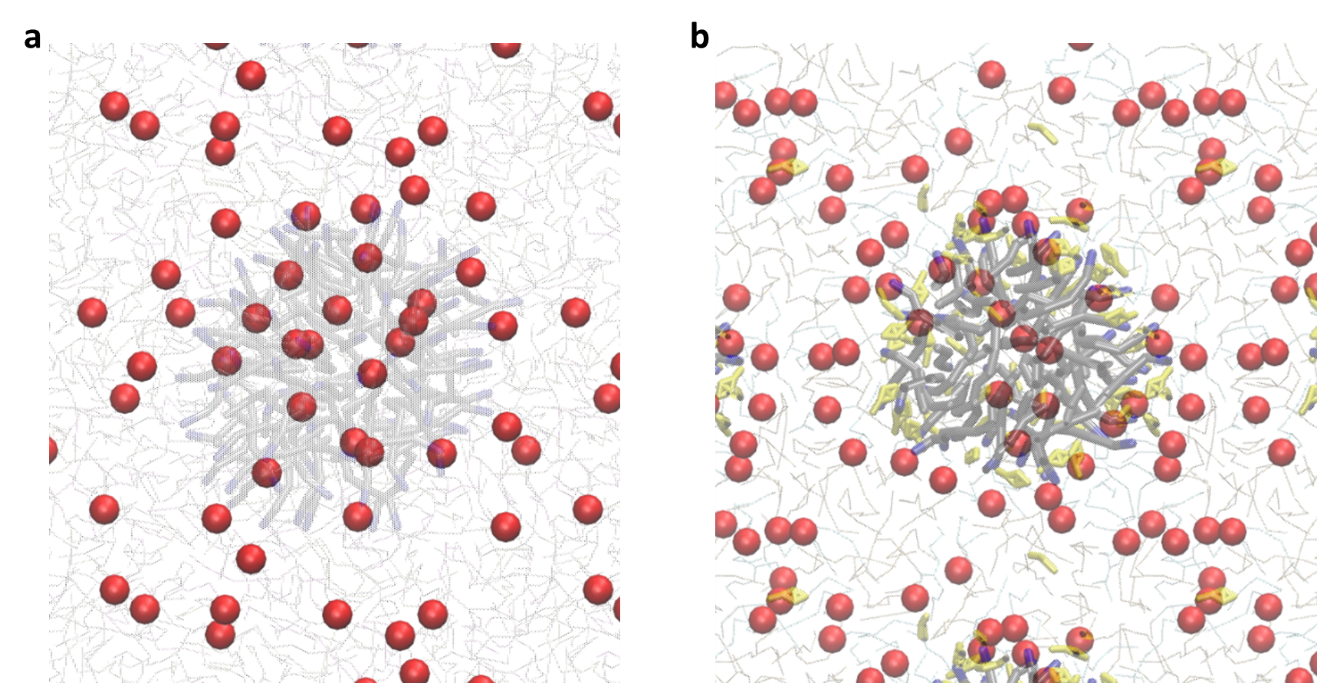


*Figure S- 15:* *Top view snapshots of a cetrimonium micelle and a CTA 4-OHcinn micelle bound to a 48%POPG-52%POPE and a 50%POPG-50%POPE bilayer, respectively (a and b).*

Figure S- 16a shows a simulation snapshot after 30 µs, regarding the interaction between 4-OH cinnamate anions and a mixed bilayer composed of 50% POPE + 50% POPG. It is noticed that the concentration of 4-OH cinnamate is lower in the upper leaflet compared to the bulk phase, which might be attributed to the electrostatic repulsion between the aromatic anions and the bilayer surface (Figure S- 16b).

**
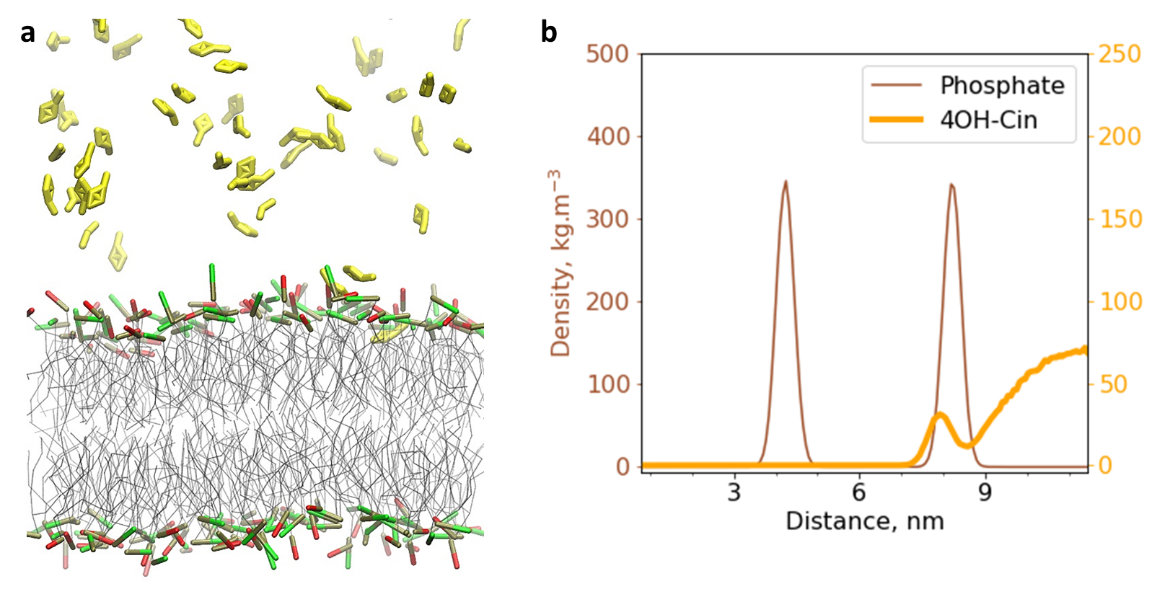
**

*Figure S- 16: Simulation snapshot taken at 30 µs regarding the interaction between 4-OH cinnamate and a mixed bilayer composed of 50% POPE + 50% POPG (a). Phosphate and 4-OH cinnamate density profiles in the bilayer normal direction (b).*

Interaction between a CTA-4OHcinn micelle and asymmetric bilayers whose lower leaflet is composed with POPE and the upper leaflet contain a mixture of POPE + 75% Re-LPS, 50% Re-LPS and 25% Re-LPS, with their respective density profiles (Figure S- 17).

**
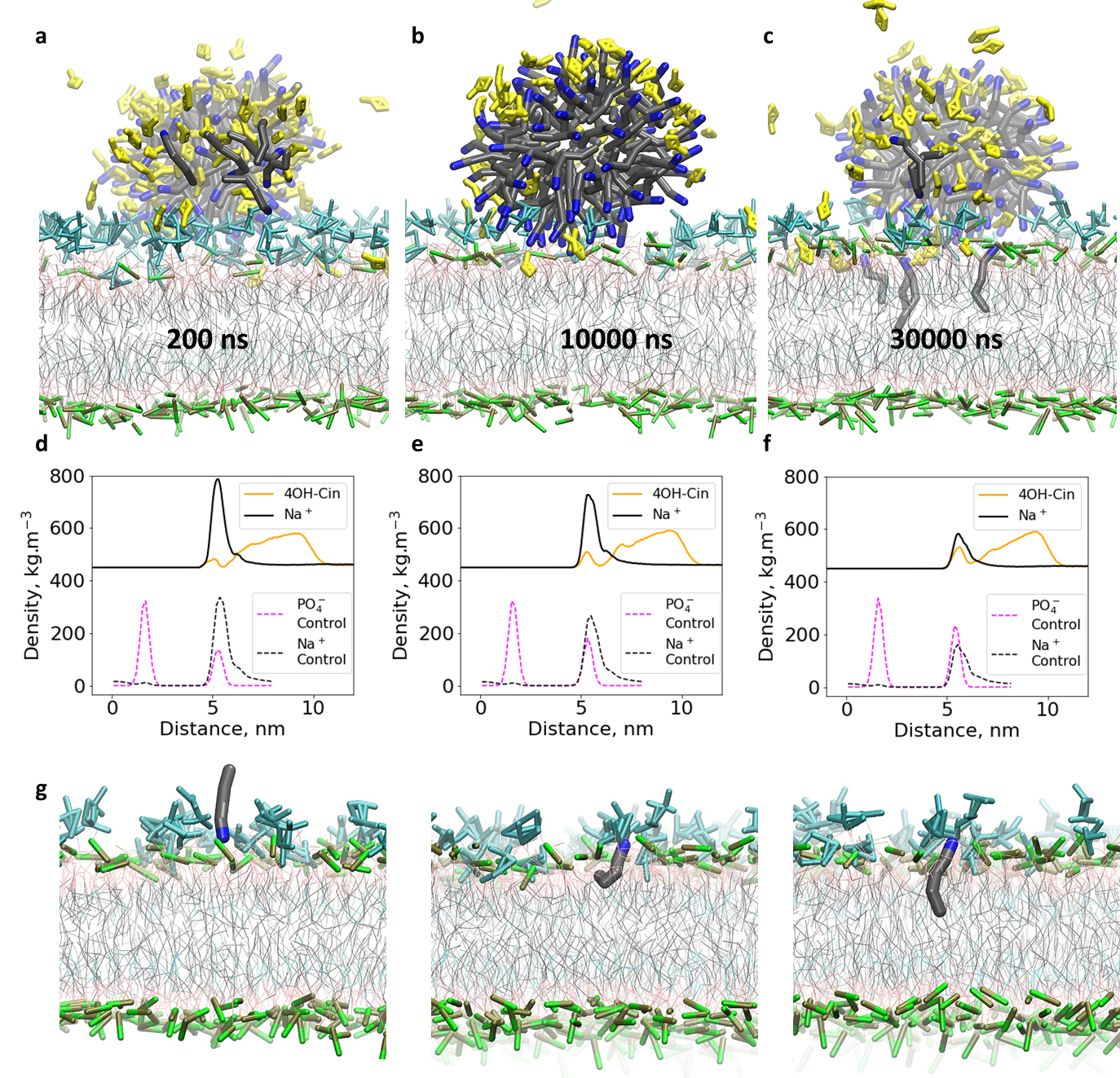
**

*Figure S- 17: Simulation snapshots of a CTA-4OHcinn micelle with asymmetric bilayers whose lower leaflet is composed with POPE and the upper leaflet contain a mixture of POPE + 75% Re-LPS, 50% Re-LPS and 25% Re-LPS (a-c). Density profile of Na counterions, 4-OH cinnamate and bilayer phosphates for a CTA-4OHcinn micelle and asymmetric bilayers composed of a mixture of POPE + 75% Re-LPS, 50% Re-LPS and 25% Re-LPS (d-f). Flip mechanism for cetrimonium insertion (g).*

Figure S- 18 shows the configuration of sodium counterions before and after micelle merging for a symmetric and an asymmetric bilayer. Notice that after micelle merging, the counterion concentration decreases considerably on the disrupted bilayer surface.


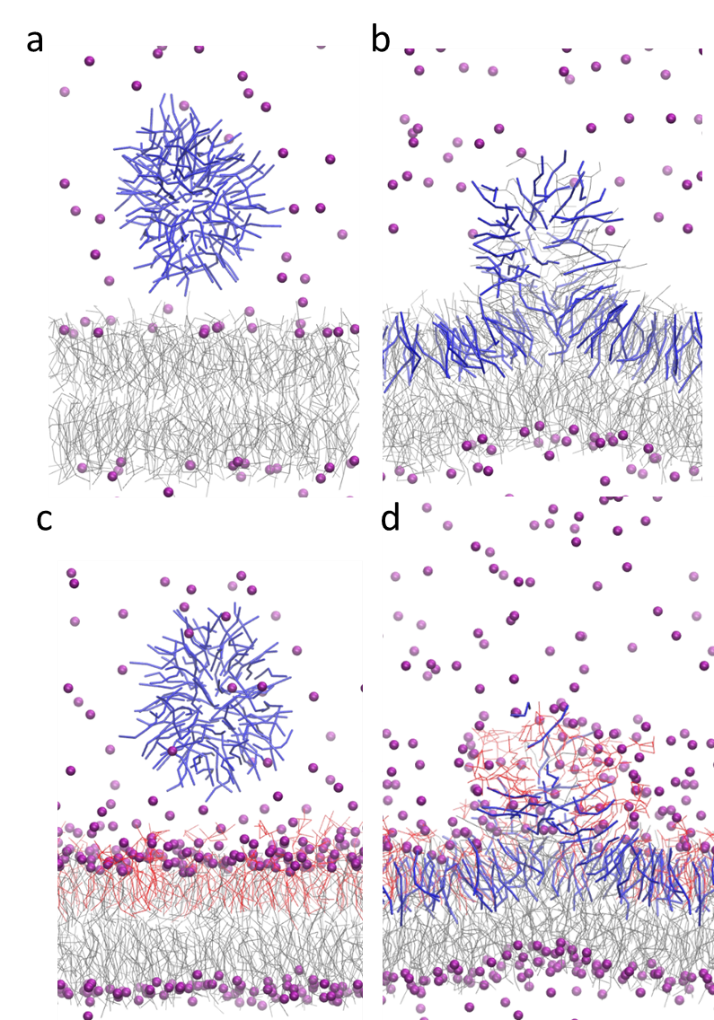


*Figure S- 18: Simulation snapshots of a cetrimonium micelle (blue) before and after merging with a symmetric bilayer (a and b, respectively) and with an asymmetric bilayer, where the red molecules represent the LPS (c and d, respectively). The purple spheres represent the sodium counterions.*

Figure S- 19 a and b shows the simulation snapshots regarding the interaction between 0.3 M CTA-4OHcinn and a two DNA sequences of 16 bases, an adenine-thymine (AT-DNA) and a cytosine-guanine (CG-DNA) pair. Figure S- 19 c and d shows the RDF for cetrimonium amine groups and 4OH-cinnamate anions taking as a reference the AT-DNA and CG-DNA backbone.


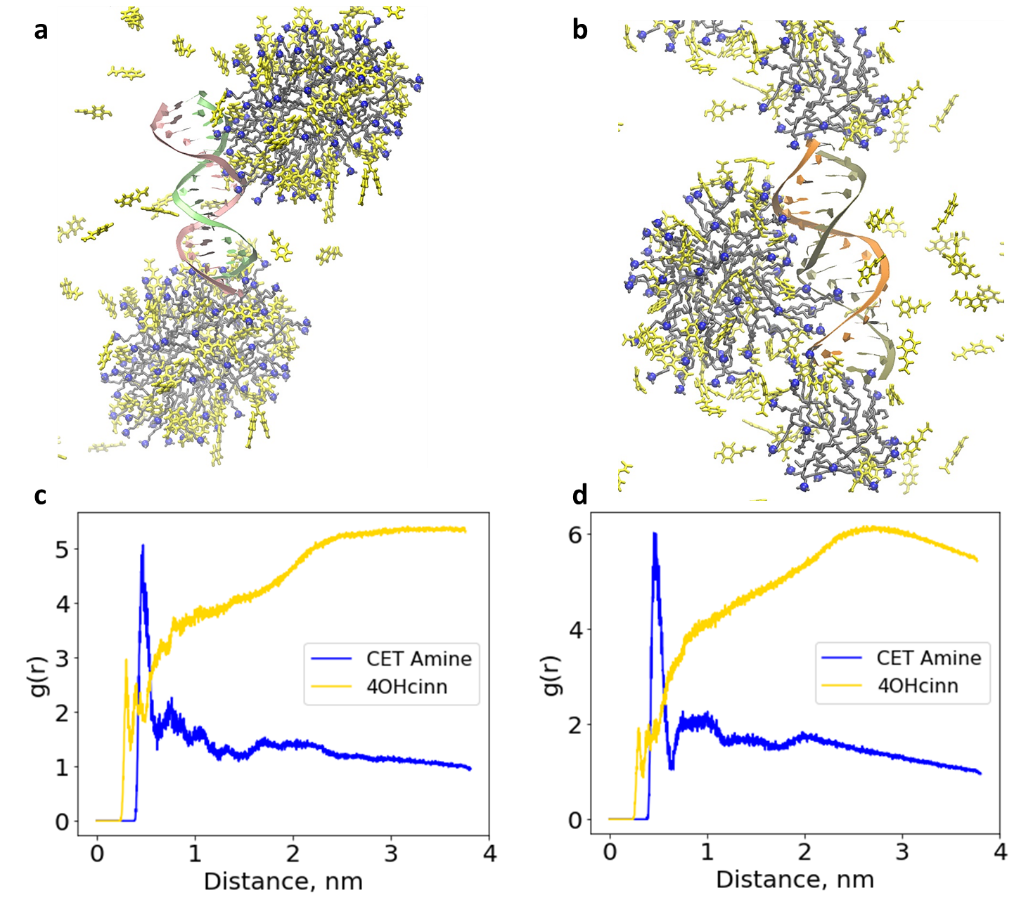


Figure S- 19: Simulation snapshots of an adenine-thymine DNA (AT-DNA) and a cytosine-guanine DNA (CG-DNA) after interaction with 0.3 M CTA-4OHcinn (a and b, respectively). RDF of the cetrimonium amine groups and 4OH-cinnamate anions taking as a reference the AT-DNA and CG-DNA backbone (b and d, respectively).

**REFERENCES**

1 Yesylevskyy, S. O., Schäfer, L. V., Sengupta, D. & Marrink, S. J. Polarizable water model for the coarse-grained MARTINI force field. *PLoS Comput. Biol.* **6**, e1000810, doi:10.1371/journal.pcbi.1000810 (2010).

2 Bussi, G., Donadio, D. & Parrinello, M. Canonical sampling through velocity rescaling. *J. Chem. Phys.* **126**, 014101, doi:10.1063/1.2408420 (2007).

3 Berendsen, H. J. C., Postma, J. P. M., Gunsteren, W. F., DiNola, A. R. H. J. & Haak, J. R. Molecular dynamics with coupling to an external bath. *J. Chem. Phys.* **81**, 3684-3690, doi:10.1063/1.448118 (1984).

4 Ingolfsson, H. I. *et al.* In Silico Modeling of Biologically Complex Membranes. *Biophys. J.* **110**, 83a (2016).

5 Sachs, J. N., Crozier, P. S. & Woolf, T. B. Atomistic simulations of biologically realistic transmembrane potential gradients. *J. Chem. Phys.* **121**, 10847-10851, doi:10.1063/1.1826056 (2004).

6 Shahane, G., Ding, W., Palaiokostas, M. & Orsi, M. Physical properties of model biological lipid bilayers: insights from all-atom molecular dynamics simulations. *J. Mol. Model.* **25**, 1-13, doi:10.1007/s00894-019-3964-0 (2019).

7 Goyal, P. S. *et al.* Shapes and sizes of micelles in CTAB solutions. *Phys. B* **174**, 196-199, doi:10.1016/0921-4526(91)90606-F (1991).

8 Imae, T., Kamiya, R. & Ikeda, S. Formation of spherical and rod-like micelles of cetyltrimethylammonium bromide in aqueous NaBr solutions. *J. Colloid Interface Sci.* **108**, 215-225, doi:10.1016/0021-9797(85)90253-X (1985).

9 Hayter, J. B. & Penfold, J. Determination of micelle structure and charge by neutron small-angle scattering. *Colloid Polym. Sci.* **261**, 1022-1030, doi:10.1007/BF01421709 (1983).

10 Soto Puelles, J. *et al.* Modelling cetrimonium micelles as 4-OH cinnamate carriers targeting a hydrated iron oxide surface. *J. Colloid Interface Sci.*, doi:10.1016/j.jcis.2021.11.139 (2021).

11 Vesenka, J., Marsh, T., Henderson, E. & Vellandi, C. The diameter of duplex and quadruplex DNA measured by scanning probe microscopy. *Scanning Microsc.* **12**, 329-342 (1998).

12 Alberts, B. *et al.* *Molecular biology of the cell*. (WW Norton & Company, 2017).

13 Hognon, C. *et al.* Molecular bases of DNA packaging in bacteria revealed by all-atom molecular dynamics simulations: The case of histone-like proteins in borrelia burgdorferi. *J. Phys. Chem. Lett.* **10**, 7200-7207, doi:10.1021/acs.jpclett.9b02978 (2019).
